# ZipA Uses a Two-Pronged FtsZ-Binding Mechanism Necessary for Cell Division

**DOI:** 10.1128/mbio.02529-21

**Published:** 2021-12-14

**Authors:** Todd A. Cameron, Daniel E. Vega, Chenfei Yu, Han Xiao, William Margolin

**Affiliations:** a Department of Microbiology and Molecular Genetics, McGovern Medical School, Houston, Texas, USA; b Department of Chemistry, Biosciences and Bioengineering, Rice University, Houston, Texas, USA; University of Nebraska Medical Center

**Keywords:** *Escherichia coli*, FtsZ, ZipA, cell division, photo-cross-linking, protein-protein interactions

## Abstract

In most bacteria, cell division is centrally organized by the FtsZ protein, which assembles into dynamic filaments at the division site along the cell membrane that interact with other key cell division proteins. In gammaproteobacteria such as Escherichia coli, FtsZ filaments are anchored to the cell membrane by two essential proteins, FtsA and ZipA. Canonically, this interaction was believed to be mediated solely by the FtsZ C-terminal peptide (CTP) domain that interacts with these and several other regulatory proteins. However, we now provide evidence of a second interaction between FtsZ and ZipA. Using site-specific photoactivated cross-linking, we identified a noncanonical FtsZ-binding site on ZipA on the opposite side from the FtsZ CTP-binding pocket. Cross-linking at this site was unaffected by the truncation of the FtsZ linker and CTP domains, indicating that this noncanonical site must interact directly with the globular core domain of FtsZ. Mutations introduced into either the canonical or noncanonical binding sites on ZipA disrupted photo-cross-linking with FtsZ and normal ZipA function in cell division, suggesting that both binding modes are important for normal cell growth and division. One mutation at the noncanonical face was also found to suppress defects of several other canonical and noncanonical site mutations in ZipA, suggesting there is some interdependence between the two sites. Taken together, these results suggest that ZipA employs a two-pronged FtsZ-binding mechanism.

## INTRODUCTION

Bacterial cell division is a highly choreographed process that brings together dozens of proteins for the formation of a division septum and separation into daughter cells (1). Central to this process is FtsZ, a homolog of eukaryotic tubulin. Treadmilling filaments of FtsZ serve to recruit and organize numerous cell division proteins into a functioning divisome in most bacterial species (2, 3). In Escherichia coli and other gammaproteobacteria, FtsZ is tethered to the cell membrane by two essential proteins, FtsA and ZipA (4, 5). All three proteins together assemble into the early proto-ring at the beginning of divisome formation, along with the nonessential FtsZ-stabilizing proteins ZapA, ZapC, and ZapD (6). As the divisome matures, and for the remainder of cell division, dynamic filaments of FtsZ continually interact with FtsA and ZipA in order to maintain a close association with the cell membrane and to organize cell division proteins at the cell envelope.

The interaction between FtsZ and its membrane anchors is essential for cell division, which fails to progress in the absence of either FtsA or ZipA (7). Although both proteins anchor FtsZ, each protein has distinct functions in cell division. FtsA, an actin homolog with an amphipathic C-terminal tail, interacts with FtsZ filaments and is required for recruitment of later divisome proteins such as FtsN, FtsEX, and FtsK. In contrast, ZipA is a monotopic membrane protein with a cytoplasmic globular domain that interacts with both monomeric and polymerized forms of FtsZ (4, 8). ZipA primarily serves as a passive membrane anchor for FtsZ, but it also interacts directly with FtsA (9, 10). This interaction with FtsA may promote disruption of FtsA miniring structures, which have been observed on lipid monolayers by electron microscopy (EM) (11). Disruption of FtsA miniring formation in this system facilitated close lateral associations between FtsZ polymers, which are probably important for transitioning the protoring into a functioning divisome (12–14).

FtsZ interacts with ZipA, FtsA, and several other cell division proteins through a small conserved C-terminal peptide (CTP) attached by a flexible linker to the globular domain of FtsZ (15). Crystallography revealed that purified FtsZ CTP binds to a hydrophobic pocket within the globular domain of ZipA (16). Alanine substitutions within the FtsZ CTP corresponding to residues I374, F377, and L378 of the full-length FtsZ were particularly effective at disrupting this interaction as assessed by surface plasmon resonance (16), consistent with earlier FtsZ truncation studies that established the importance of the FtsZ CTP (17–19).

**FIG 1:**
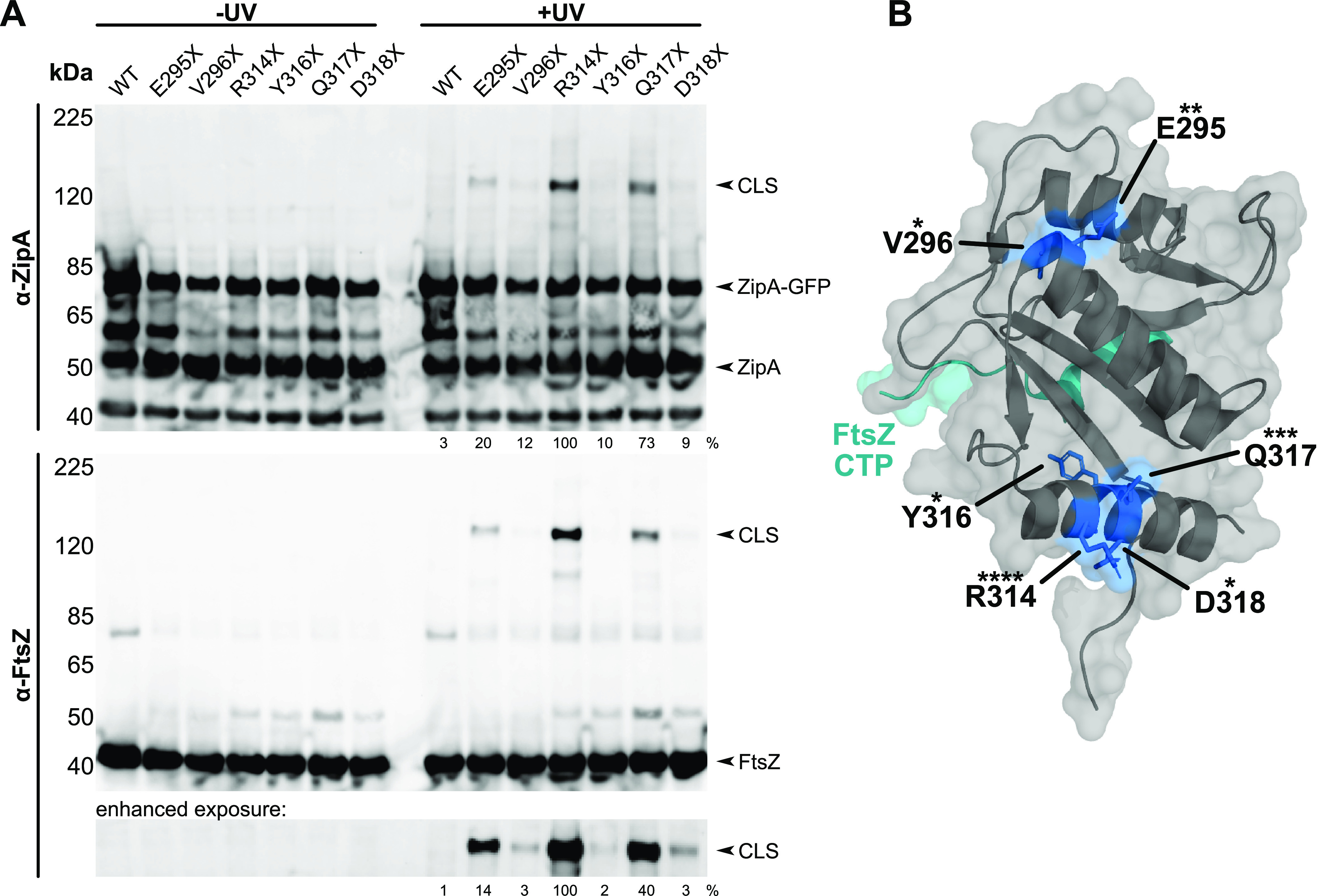
ZipA cross-links FtsZ at noncanonical residues. (A) Anti-ZipA (α-ZipA) and anti-FtsZ (α-FtsZ) Western blots of cell lysates of strain WM1074 carrying pUltra-pBpF, pKG110-FtsZ, and pDSW210-ZipA-GFP or derived constructs containing indicated Bpa substitutions in ZipA. CLS containing ZipA-GFP and FtsZ readily formed for residues R314X, Q317X, and E295X, but can be observed for all constructs with enhanced exposure (bottom panel). Cross-linking efficiency relative to ZipA(R314X)-GFP is indicated below +UV lanes and was normalized by total protein levels and ZipA-GFP expression for each strain. (B) Crystal structure of ZipA (gray) bound to the FtsZ CTP (teal) (16) annotated with each noncanonical cross-linking residue (blue). Asterisks indicate relative cross-linking strength with more asterisks indicated higher cross-linking strength.

**FIG 2:**
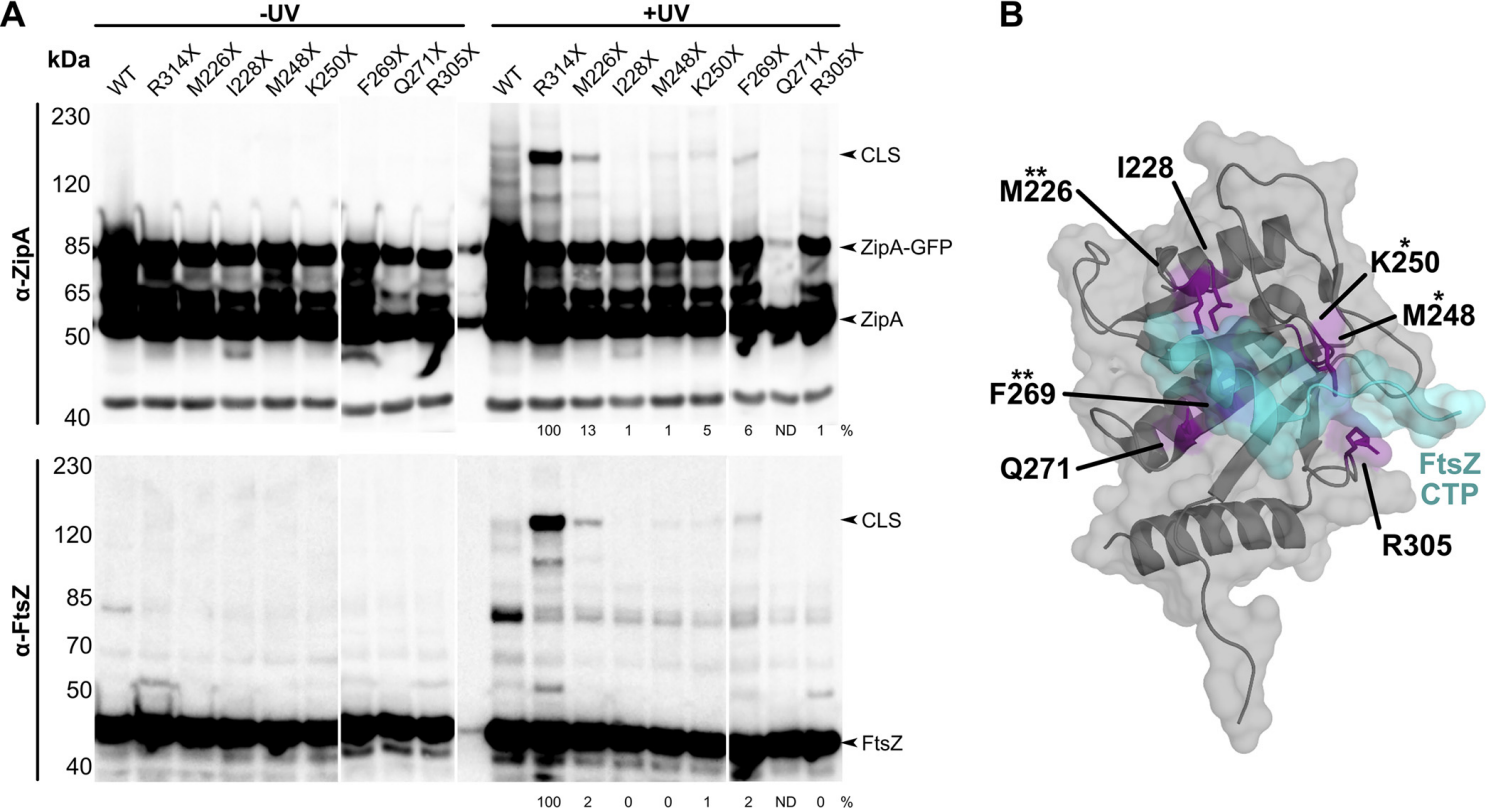
ZipA cross-links FtsZ at canonical residues. (A) Anti-ZipA and anti-FtsZ Western blots of WM1074 cell lysates carrying pUltra-pBpF, pKG110-FtsZ, and pDSW210-ZipA-GFP or derived constructs containing indicated Bpa substitutions in ZipA. Canonical site substitutions M226X and F269X produced CLS of moderate intensity compared to the noncanonical site R314X. Cross-linking efficiency relative to ZipA(R314X)-GFP on each blot is indicated below +UV lanes and was normalized by total protein levels and ZipA-GFP expression for each strain. ND, not determined. Each panel shows a composite of two blots. (B) Crystal structure of ZipA (gray) bound to the FtsZ CTP (teal) (16), rotated 180° around a vertical axis compared with the structure in Fig. 1, annotated with each canonical residue tested for cross-linking (purple). Asterisks indicate cross-linking strength relative to the noncanonical site R314X.

However, much of the ∼140-residue globular domain of ZipA is outside the hydrophobic pocket bound to the FtsZ CTP in the crystal structure (see Fig. 1B and 2B), suggesting that additional interactions may occur between ZipA and FtsZ or other divisome proteins. In support of this hypothesis, the interaction between ZipA and the FtsZ CTP was about 100 times weaker than with full-length FtsZ (16). Although a second study showed that oligomerization of FtsZ plays a significant role in strengthening ZipA’s apparent interaction with full-length FtsZ, the possibility of a secondary binding site could not be ruled out (8). Moreover, a recent cryo-EM structure of full-length ZipA in complex with FtsZ in lipid nanodiscs (20) showed that the globular domains of both proteins were closely associated, hinting at additional interactions beyond the canonical FtsZ CTP-binding site.

Here, by utilizing site-specific photoactivated cross-linking and genetic approaches, we have identified a novel binding interface on ZipA that lies distal to the canonical FtsZ-binding site and, importantly, does not bind the FtsZ CTP. Both sites facilitate FtsZ binding, which can be disrupted by introducing mutations to ZipA at either site. As with mutations within the canonical site, mutations within the noncanonical site of ZipA also result in a reduction or complete loss of function *in vivo*. Our results suggest a dual interaction between FtsZ and ZipA that may be important for their function.

## RESULTS

### Novel interaction between FtsZ and several residues near the extreme ZipA C terminus.

In a search to find the domain of ZipA that contacts FtsA (10), we focused on a region in the globular domain of ZipA distal from the reported FtsZ CTP-binding site (16). The most attractive area for this was the extreme C terminus of ZipA, which harbors two alpha helices of unknown function. We engineered amber stop codons throughout these helices for use in *p*-benzoyl-l-phenylalanine (Bpa) cross-linking experiments, using a ZipA-green fluorescent protein (GFP) expression plasmid. When ZipA-GFP with an individual amber codon is synthesized in cells expressing the cognate tRNA and exposed to Bpa in the medium, Bpa will be inserted at the amber codon by the tRNA, resulting in a ZipA protein with Bpa at a specific residue, hereafter designated with “X”. Subsequent UV cross-linking of cells producing each ZipA-Bpa can then identify residues within these helices that contact another protein within 3.1 Å of the benzophenone moiety ketone oxygen, or about 3 to 9 Å from the polypeptide backbone (21). Cross-linking by benzophenones occurs with many amino acids, although they favor proline, methionine, threonine, arginine, and serine (22).

We found that several Bpa-substituted residues in these helices, including E295, V296, R314, Y316, Q317, and D318, gave rise to detectable high-molecular-weight cross-linked species (CLS) after UV irradiation when probed with anti-ZipA on immunoblots (Fig. 1A, top). Although no CLS were detectable on immunoblots probed with anti-FtsA (data not shown), we were surprised to find that probing with anti-FtsZ detected strong CLS for several Bpa constructs, particularly ZipA(R314X)-GFP, with Bpa at R314 (Fig. 1A, bottom). Other Bpa-substituted residues in this region of ZipA that generated FtsZ-containing CLS included E295X and Q317X, but the intensities of the CLS bands for these were significantly weaker than the R314X band, and the intensities of the CLS bands for V296X, Y316X, and D318X were even weaker (Fig. 1A). Interestingly, the strongest CLS bands correlated with residues on these helices that faced the surface, away from the known FtsZ-binding site (Fig. 1B). Although initial cross-linking experiments were done with cells expressing native levels of FtsZ, we obtained greater sensitivity when we used cells expressing FtsZ from a compatible plasmid, which were used for the experiments shown in Fig. 1.

To rule out the possibility that the GFP fusion to ZipA was causing artifactual cross-linking, we also tested ZipA(R314X) with no GFP fusion and observed a clear CLS band that migrated significantly faster than the CLS from the ZipA(R314X)-GFP fusion (see Fig. S1 in the supplemental material). These results indicated that the GFP fusion did not affect the cross-linking from ZipA(R314X).

10.1128/mbio.02529-21.1 FIG S1 Noncanonical cross-linking at R314X is unaffected by a GFP fusion to the C terminus of ZipA. Cell lysates of strain WM1074 containing pUltra-pBpF and pKG110-FtsZ, plus pDSW210-ZipA, pDSW210-ZipA-GFP, or derived R314X constructs were separated on 7.5% TGX precast gels (BioRad) and assessed by Western blotting after SDS-PAGE. CLS containing ZipA or ZipA-GFP and FtsZ readily formed upon UV treatment. CLS band intensity relative to ZipA(R314X)-GFP is indicated below the +UV lanes and normalized by total protein levels. Download FIG S1, PDF file, 0.1 MB. Copyright © 2021 Cameron et al. 2021 Cameron et al. https://creativecommons.org/licenses/by/4.0/ This content is distributed under the terms of the Creative Commons Attribution 4.0 International license.

### FtsZ cross-linking to ZipA(R314X) is stronger than to ZipA residues at the previously characterized FtsZ-binding interface.

Our results were unexpected because the previously reported “canonical” FtsZ-binding site on ZipA is ∼20 Å from several residues such as R314 that we newly identified by cross-linking (Fig. 1B). We wanted to confirm that our cross-linking method could also detect canonical binding and to compare the relative intensities between canonical and noncanonical cross-links. For this purpose, we chose several ZipA residues known to make direct contacts with the FtsZ CTP in the cocrystal: M226, I228, M248, K250, F269, and R305 (Fig. 2B). We replaced these residues with amber stop codons in our ZipA-GFP expression construct and measured the degree of UV cross-linking to FtsZ when these residues were replaced with Bpa.

After UV irradiation and probing with anti-FtsZ or anti-ZipA, we detected very weak high-molecular-weight CLS bands for Bpa at M248 and K250, whereas Bpa at I228, K250, and R305 resulted in barely detectable or undetectable CLS bands (Fig. 2A). The Q271X construct was unstable. Notably, Bpa at F269 [ZipA(F269X)-GFP] resulted in a stronger CLS band, and Bpa at M226 [ZipA(M226X)-GFP] gave the strongest CLS signal for these canonical site residues. Nonetheless, the noncanonical cross-linking from R314X conferred by far the strongest CLS signals, which may reflect a generally more favorable cross-linking environment in the noncanonical ZipA-FtsZ interface compared with the canonical site. These results indicate that many of the canonical binding site residues reported to make direct contacts with FtsZ do indeed cross-link, which validates our methods and confirms that the residues that interacted in the cocrystal do indeed interact *in vivo*.

### Importance of R314 for ZipA function.

**FIG 3:**
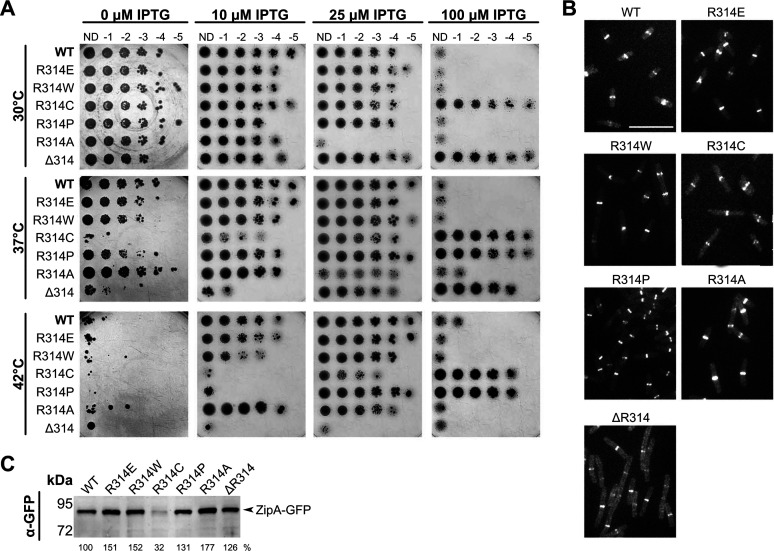
ZipA R314 is important for ZipA function. (A) Serial dilutions of strain WM5337 (*zipA1*) containing pDSW210-ZipA-GFP or derived mutants were spotted on plates containing 0, 10, 25, or 100 μM IPTG and grown at 30°C, 37°C, or 42°C. Growth is permissive at 30°C and restrictive approaching 42°C. Only the ZipA(ΔR314)-GFP failed to grow under any level of IPTG at 42°C. (B) Representative images of ZipA-GFP localization in the same strains. Cultures were grown for 2 h at 30°C with 10 μM IPTG and imaged by fluorescence microscopy. While all constructs localized to mid-cell rings, ZipA(ΔR314)-GFP exhibited additional fluorescence throughout the cell. Bar, 7 μm. (C) Expression levels of each ZipA-GFP construct were assessed by Western blotting. All constructs were stable except for ZipA(R314C)-GFP. Band intensity relative to the parent construct is indicated below each lane.

To explore the role of noncanonical interactions in ZipA function, we first determined whether changing the strongest cross-linking residue, R314, to other residues resulted in a phenotype. Surprisingly, we found that ZipA-GFP proteins in which R314 was changed to E, W, C, P, or A were all able to complement the *zipA1* thermosensitive allele at 42°C (Fig. 3A). R314W and the charge-switched residue change R314E behaved identically to wild-type (WT) ZipA, whereas R314P and R314C needed higher levels of isopropyl-β-d-thiogalactopyranoside (IPTG) induction to complement and were less toxic than WT ZipA at lower temperatures, indicating a modest loss of function (Fig. 3A). Consistent with this, R314C was significantly less stable at 30°C compared with WT ZipA and the other mutants. R314A was more toxic than WT ZipA, perhaps because of higher stability. In any case, we can conclude that residue 314 does not need to be an arginine, which is reasonable given that replacing it with a Bpa permits robust cross-linking with FtsZ.

These results suggest that multiple residues of ZipA near R314 are involved in noncanonical binding to FtsZ, potentially as part of a binding face that encompasses one or more alpha helices. To test this idea, we deleted R314 entirely, which would be expected to perturb alpha helix 3 (16). Indeed, we found that ZipA(ΔR314)-GFP, despite being made as a stable protein (Fig. 3B), had a notable loss-of-function phenotype. It was nontoxic at 30°C, needed higher induction levels to complement *zipA1* at 37°C, and was completely nonfunctional at 42°C (Fig. 3A). ZipA(ΔR314)-GFP localized to Z rings at 30°C, but there was higher background fluorescence throughout the cytoplasm than with ZipA-GFP or the single residue changes (Fig. 3C), implying a weakened ability to interact with FtsZ.

### The noncanonical region of ZipA interacts with the core globular domain of FtsZ.

In the established model for divisome assembly, FtsA and ZipA, through the canonical binding pocket, tether FtsZ to the membrane by binding to the FtsZ CTP. An obvious question prompted by our discovery of noncanonical interactions with FtsZ mediated by R314 and other nearby residues of ZipA was whether they required the FtsZ CTP. To address this, we used FtsZ(1–316), a derivative of FtsZ deleted for the C-terminal 67 residues comprising the flexible linker and the conserved CTP. FtsZ(1–316) was coexpressed in cross-linking experiments with ZipA(R314X)-GFP and ZipA(M226X)-GFP.

**FIG 4:**
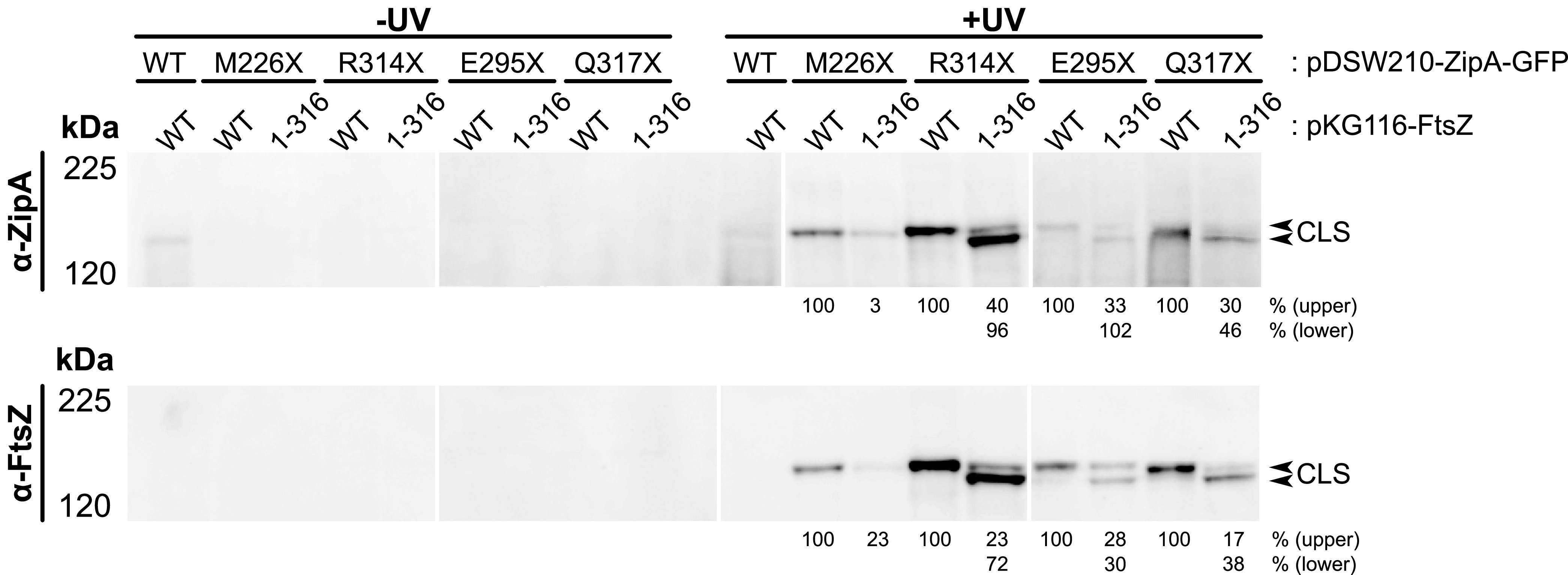
Noncanonical ZipA interaction occurs with the FtsZ globular domain. Bpa-mediated cross-linking between ZipA-GFP and FtsZ was assessed in strain WM1074 containing pUltra-pBpF and the indicated pKG116-FtsZ and pDSW210-ZipA-GFP constructs. Cell lysates of strains expressing either full-length FtsZ or only the FtsZ globular domain (1-316) in combination with ZipA-GFP carrying Bpa substitutions at canonical or noncanonical sites were separated by 6% Tris-glycine SDS-PAGE. ZipA-GFP constructs with Bpa at noncanonical binding sites formed two CLS bands containing ZipA-GFP and FtsZ; the upper band represents cross-linking with natively expressed full-length FtsZ, whereas the lower band represents direct cross-linking with the FtsZ(1-316) globular domain. Cross-linking efficiency is shown below +UV lanes and indicates the relative intensity of each CLS compared to the CLS obtained with pKG116-FtsZ and the given ZipA construct. Calculations were normalized by total protein levels and ZipA-GFP expression for each strain. Each panel shows a composite of two blots.

As expected, ZipA(M226X)-GFP did not cross-link with FtsZ(1–316), as M226 is part of the canonical binding site that interacts with the FtsZ CTP (Fig. 4). In contrast, ZipA(R314X)-GFP interacted robustly with FtsZ(1–316), strongly suggesting that R314X, and thus noncanonical binding, requires the core globular domain of FtsZ and not the C-terminal peptide (Fig. 4). We also tested whether other residues in the noncanonical domain of ZipA could interact with FtsZ(1–316) and confirmed that they indeed resulted in strong CLS bands (Fig. 4). These results strongly suggest that, contrary to established models, the noncanonical interaction between ZipA and FtsZ involves the conserved core of FtsZ.

### ZipA(F269S) is a nonfunctional mutant in the canonical site.

ZipA is toxic when overproduced. Although the molecular mechanism for this toxicity is not clear, it is likely linked to ZipA’s ability to bind to FtsZ. Therefore, to conduct an unbiased search for mutants of ZipA potentially defective in interacting with FtsZ through canonical or noncanonical binding, we screened for ZipA mutants that are no longer toxic when overproduced. Notably, one of the mutants we isolated contained F269S, a change in a critical canonical residue. Isolation of such a mutant was not surprising, as F269 makes direct contact with FtsZ in the cocrystal and inactivating this interaction would be expected to weaken ZipA-FtsZ binding via the canonical site. Nevertheless, this mutant was potentially useful for uncoupling canonical from noncanonical interactions.

**FIG 5:**
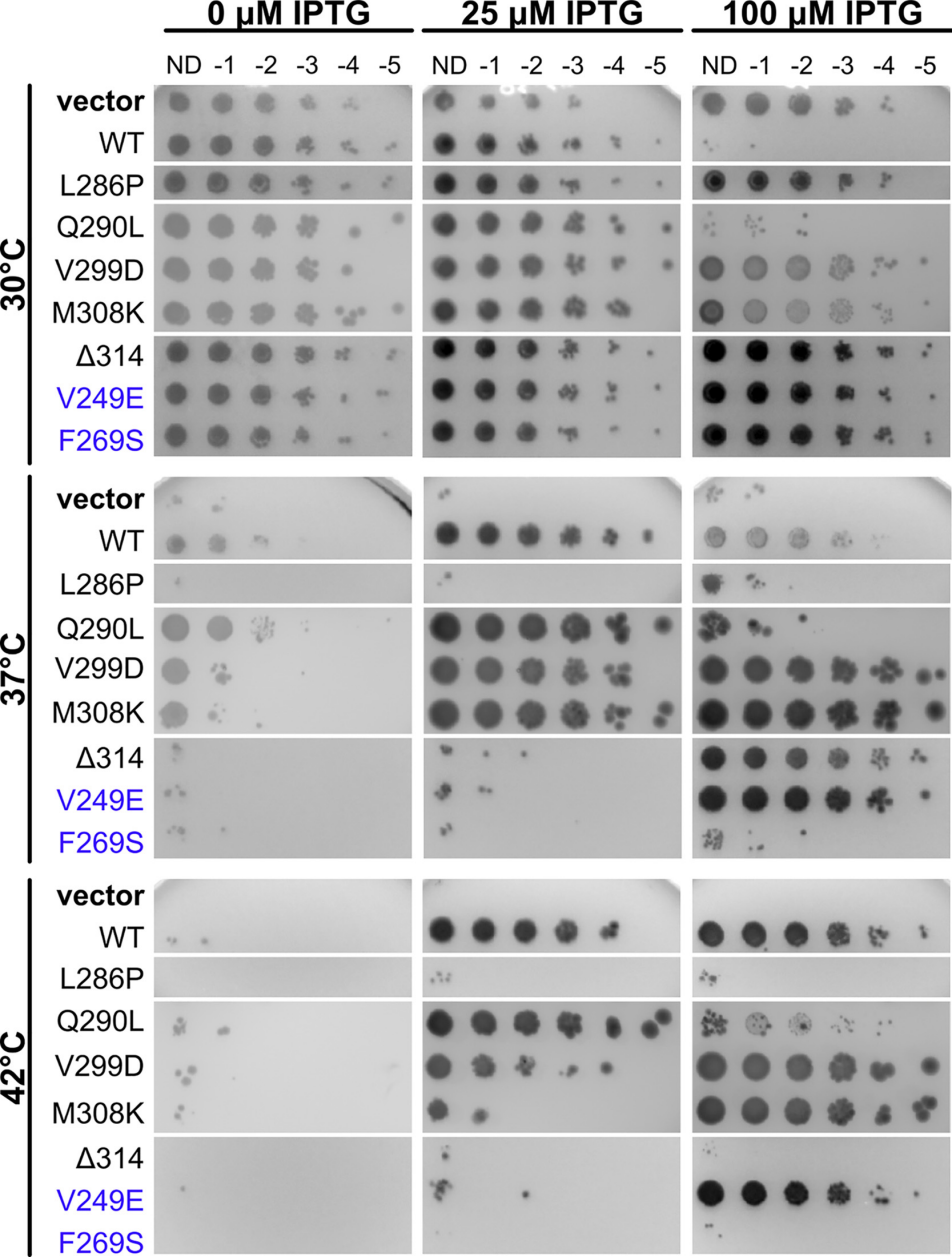
Residue changes on the noncanonical face of ZipA disrupt ZipA function. Cultures of WM5337 (*zipA1*) transformed with pDSW210-GFP (vector), pDSW210-ZipA-GFP, or derived constructs were serially diluted and spotted on plates containing 0, 25, or 100 μM IPTG and grown at permissive (30°C) or restrictive (37° and 42°C) temperatures. Canonical face residue changes are indicated with purple labels. Each panel shows a composite image of several plates. Although all strains grew well at 30°C, most either did not grow or required higher levels of IPTG induction when grown at 42°C.

To check whether F269S affects ZipA function, we first tested ZipA(F269S)-GFP for its ability to complement *zipA1*(Ts) and localize to Z rings. As expected, cells with the *zipA1* allele but only empty pDSW210 plasmid vector grew normally at 30°C but were inviable at 37°C or 42°C. When expressed from the IPTG-inducible but weakened *Trc* promoter on pDSW210 at 42°C, ZipA-GFP levels were insufficient for viability at 0 μM IPTG, but increased expression at 25 or 100 μM permitted full viability (Fig. 5, WT rows). ZipA-GFP (or ZipA) becomes toxic at higher expression levels, particularly at lower temperatures where functional ZipA1 protein is also present. As a result, at 37°C the best viability for WT ZipA-GFP was at 25 μM IPTG, high enough for complementation but not high enough to inhibit cell division significantly (Fig. 5, WT rows).

In contrast, ZipA(F269S)-GFP was not toxic when induced at high levels at 30°C and failed to complement the *zipA1* mutant at any induction level (Fig. 5, F269S rows). In addition, attempts to transduce a null allele of *zipA* (Δ*zipA*::*kan*) into a strain expressing ZipA(F269S)-GFP were unsuccessful (see also below). These results indicate that the F269S residue change severely disrupts ZipA function in cell division. Consistent with this, ZipA(F269S)-GFP failed to localize to Z rings, instead localizing mainly to the cell membrane and occasional foci distributed along the cell length (Fig. S2).

10.1128/mbio.02529-21.2 FIG S2 Effects of ZipA mutations on ZipA-GFP localization. Shown are representative images of cells expressing pDSW210-ZipA-GFP or derived constructs. ZipA(F269S)-GFP was expressed in strain WM5337 (*zipA1*), and all other plasmids were expressed in a MG1655 *ΔzipA*::*aph* background. Cultures were either grown for 2 h at 30°C or grown for 1 h at 30°C and then shifted to 42°C for 1 h. Most constructs retain the ability to localize to mid-cell rings even under restrictive growth conditions, but localization of ZipA-GFP carrying the F269S or L286P residue changes was severely disrupted. Canonical variants are labeled in blue. Download FIG S2, PDF file, 0.2 MB. Copyright © 2021 Cameron et al. 2021 Cameron et al. https://creativecommons.org/licenses/by/4.0/ This content is distributed under the terms of the Creative Commons Attribution 4.0 International license.

### F269S significantly weakens canonical interactions between ZipA and the FtsZ CTP.

To test the ability of ZipA(F269S)-GFP to interact directly with the FtsZ CTP, we asked whether a Bpa incorporated into the CTP could photo-cross-link with ZipA, i.e., in the reverse direction from our results above. We chose FtsZ residue Y371 to label with Bpa as it is aromatic, which is similar to the Bpa replacing it, and directly contacts ZipA residues M248, V249, and R305 in the cocrystal (16). We then compared the ability of ZipA-GFP and ZipA(F269S)-GFP to be cross-linked by FtsZ(Y371X). By analogy to our experiments described above, we expressed FtsZ(Y371X) from a plasmid along with ZipA-GFP derivatives carrying mutant alleles in cells expressing native FtsZ and ZipA in order to maintain normal cell division.

**FIG 6:**
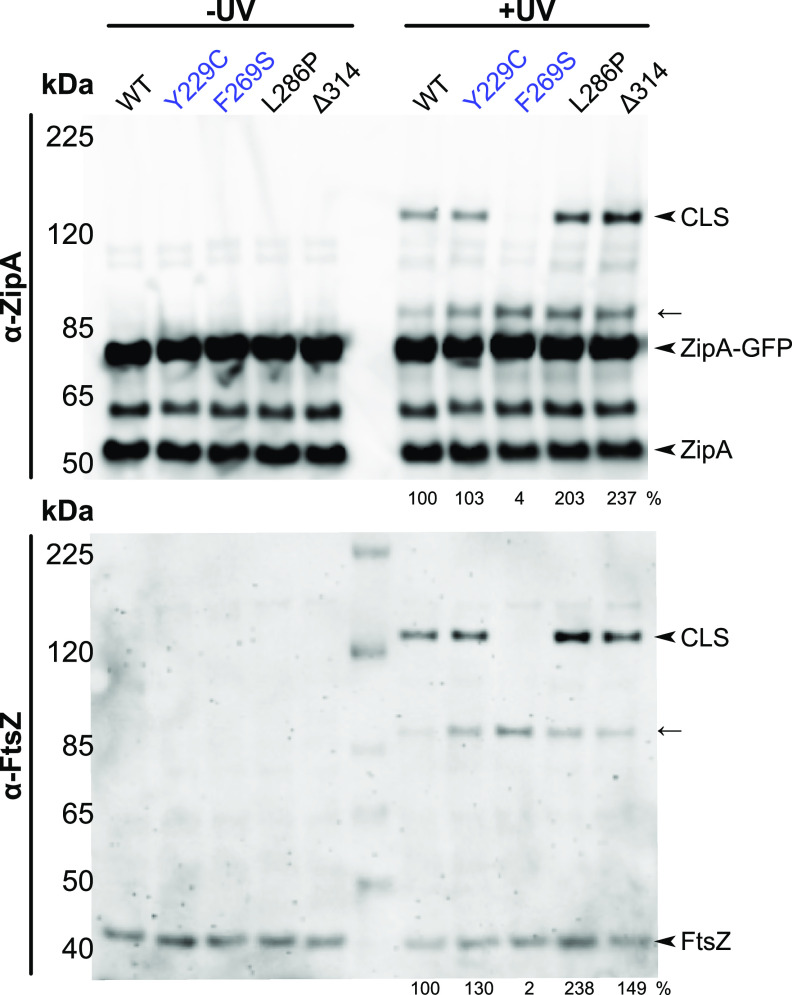
The FtsZ CTP cross-links with ZipA noncanonical site mutants. Bpa-mediated cross-linking between ZipA-GFP and FtsZ(Y371X) was assessed in strain WM1074 containing pUltra-pBpF, pKG116-FtsZ, and pDSW210-ZipA-GFP or derived mutants by Western blot analysis. FtsZ(Y371X) efficiently cross-linked ZipA-GFP carrying noncanonical binding site mutations, yielding CLS containing both proteins. The arrow indicates probable CLS formed between FtsZ(Y371X) and native ZipA. Mild (Y229C) and severe (F269S) residue changes in the canonical site of ZipA-GFP are highlighted in purple. Cross-linking efficiency relative to ZipA-GFP on each blot is indicated below +UV lanes and was normalized by total protein levels and ZipA-GFP expression for each strain.

As expected, we detected clear cross-linking between FtsZ(Y371X) and ZipA-GFP after probing immunoblots with anti-ZipA or anti-FtsZ (Fig. 6). Native ZipA was also cross-linked in each sample, serving as an internal control. Strikingly, however, no cross-linking was detected between FtsZ(Y371X) and ZipA(F269S)-GFP, although cross-linking was normal with the native ZipA in the same sample (Fig. 6). These results demonstrate that the F269S residue change strongly inhibits canonical interactions with the FtsZ C terminus.

### Effects of altering additional canonical or noncanonical residues.

We then asked whether other residue changes in or near the canonical region might also perturb ZipA function and/or ability to cross-link with FtsZ. Our screen for nontoxic alleles of ZipA yielded several independent mutants, all of which had multiple residue changes. On the basis of the residue change and its location, we selected several single residue changes for reconstruction by site-directed mutagenesis and further analysis. After incorporating these single changes into ZipA-GFP and introducing pDSW210-ZipA-GFP derivatives into the *zipA1* mutant strain, we found that half of them were less toxic than WT ZipA at the permissive temperature (Fig. 5, 30°C; also see Table S1 in the supplemental material). At 42°C, 5 of the 11 new alleles were compromised, including V249E, L286P, Q290L, V299D, and M308K (Table S1); they were either mostly inviable at any IPTG induction level (L286P) like F269S and ΔR314 or required higher IPTG induction (100 μM) for viability (V249E, V299D, and M308K). L286P was the most severely deficient, as it was as inviable as F269S at 37°C or 42°C. The exception in this group was Q290L, which appeared to primarily exacerbate ZipA overexpression toxicity at all temperatures. All these residues are located a significant distance from the canonical binding site except for V249, which directly contacts the FtsZ CTP as mentioned in the previous section. To summarize, the residue changes in increasing order of functionality were F269S < L286P < ΔR314 < V249E < M308K < V299D < Q290L, with F269S and V249E being the only two that map to the canonical binding site.

10.1128/mbio.02529-21.8 TABLE S1 ZipA mutations reconstructed for confirmatory analysis. Download Table S1, DOCX file, 0.01 MB. Copyright © 2021 Cameron et al. 2021 Cameron et al. https://creativecommons.org/licenses/by/4.0/ This content is distributed under the terms of the Creative Commons Attribution 4.0 International license.

To further test the functionality of these mutants, we attempted to introduce a *ΔzipA*::*kan* allele into strains at 30°C carrying the same plasmids expressing the ZipA mutants fused to GFP. Consistent with their viabilities on spot plates, the WT ZipA-GFP and all the mutants could be transduced except for F269S, and cells expressing ZipA-GFP carrying ΔR314 or L286P as the sole copy of ZipA were filamentous at higher temperatures (Fig. S2). At 42°C, these mutant ZipA-GFPs showed a mixture of normal fluorescent bands at division sites and abnormal punctate localization. In contrast, the other mutant ZipA-GFPs mostly localized as sharp bands at division sites, even at 42°C.

**FIG 7:**
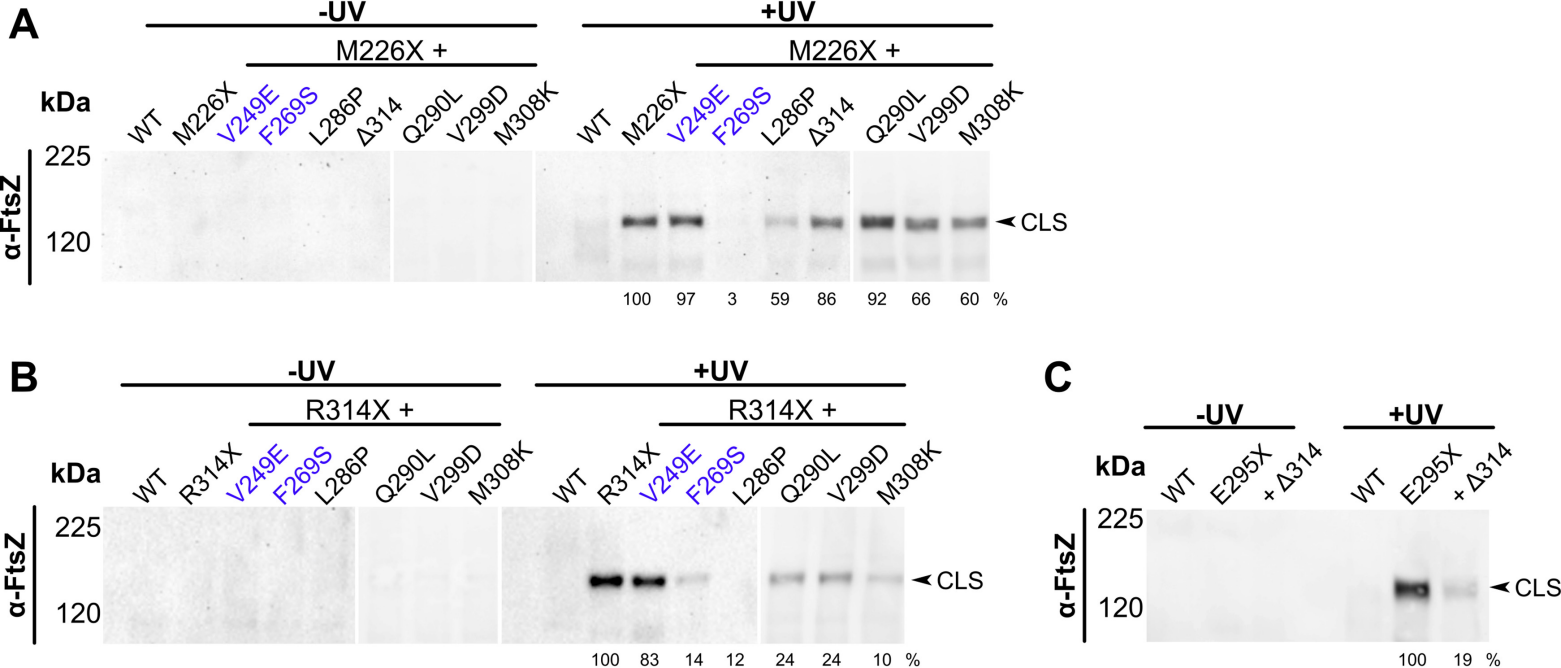
ZipA mutations disrupt canonical and noncanonical cross-linking with FtsZ. Cell lysates of strain WM1074 carrying pUltra-pBpF, pKG110-FtsZ, and pDSW210-ZipA-GFP or derived constructs containing indicated mutations were assessed by Western blotting. (A to C) Canonical binding at ZipA M226X (A) or noncanonical binding at ZipA R314X (B) or ZipA E295X (C) was assessed by measuring CLS formation alone or in combination with the indicated canonical (purple) or noncanonical (black) binding mutations. Most noncanonical mutants preferentially reduced noncanonical cross-linking. Cross-linking efficiency relative to the control is indicated below +UV lanes and was normalized by total protein levels and ZipA-GFP expression for each strain. Each panel in panels A and B shows a composite of two blots.

We next determined whether these residue changes interfered with ZipA-FtsZ cross-linking, either through the canonical site (M226X) or the noncanonical site (R314X), and whether effects on cross-linking correlated with functionality. These experiments were generally consistent with the functionalities described above. First, the canonical residue change with the most severe loss of viability, F269S, abolished canonical cross-linking with FtsZ (Fig. 7A), in agreement with data described in Fig. 6 using FtsZ(Y371X) as a cross-linking donor. Second, F269S significantly reduced, but did not eliminate, noncanonical cross-linking with FtsZ (Fig. 7B). Notably, the other canonical residue change, V249E, did not affect cross-linking from either M226X or R314X, perhaps because ZipA(V249E)’s deleterious effects are observed only at higher temperatures and not at the lower temperatures used for the cross-linking experiments.

The other noncanonical residue changes had more significant effects on cross-linking with FtsZ. The most severe noncanonical mutant, L286P, had the opposite effect of F269S, eliminating noncanonical cross-linking and significantly reducing canonical cross-linking. However, changing L286 to a proline may globally destabilize the ZipA protein, as much lower levels of the un-cross-linked protein were observed from both M226X L286P and R314X L286P constructs. In contrast, the ΔR314 mutant had a small effect on canonical cross-linking, yet it dramatically decreased cross-linking at the E295X noncanonical site (Fig. 7C). The noncanonical alleles with milder viability phenotypes, Q290L, V299D, and M308K (Fig. 5), also had significant effects on noncanonical cross-linking. Although they all retained near-normal canonical cross-linking with FtsZ through M226X (Fig. 7A), noncanonical cross-linking through R314X was significantly reduced (Fig. 7B). Overall, the cross-linking data are consistent with the viability data, with severe or mild canonical site disruptions (F269S or V249E, respectively) having severe or mild effects on viability and canonical cross-linking through M226X, and noncanonical site disruptions reducing viability and noncanonical cross-linking through R314X. It is not surprising that F269S blocks canonical interactions with FtsZ, but it is intriguing that this residue change also attenuates noncanonical interactions, suggesting a degree of interdependence between the two putative FtsZ-binding interactions.

To independently validate that our cross-linking data reflected the degree of ZipA-FtsZ interactions, we cloned the FtsZ-binding domain of ZipA (residues 189 to 328) containing the mutants into pLexA vectors for analysis in the yeast two-hybrid system in combination with pACT-FtsZ. Interaction between pACT-FtsZ and pLexA-ZipA yielded ∼210 Miller units of beta-galactosidase activity, compared to ∼4 units from the pACT vector plus pLexA-ZipA (Fig. S4), indicating that the assay can detect ZipA-FtsZ interactions as we showed previously for FtsA-FtsZ interactions (19). The data from Fig. S4 indicate that the ZipA canonical site disruptions F269S, Y229C (see below), and V249E were more defective in binding FtsZ than the individual noncanonical site disruptions, which were in turn more defective than WT ZipA. Except for the Y229C, L286P, and Q290L alleles, the strengths of the two-hybrid interactions generally correlate with the intensity of CLS bands.

10.1128/mbio.02529-21.4 FIG S4 ZipA mutants disrupt FtsZ interactions in yeast two-hybrid assays. Liquid beta-galactosidase assays were performed with yeast cotransformed with pACT2.2 (green bars) or pACT2.2-FtsZ (blue bars) and pLexA-ZipA-GFP or derived plasmids carrying the indicated *zipA* mutations. Bars indicate means and standard errors of three replicates. Mutations at canonical FtsZ-binding sites are labeled in purple text. Download FIG S4, PDF file, 0.2 MB. Copyright © 2021 Cameron et al. 2021 Cameron et al. https://creativecommons.org/licenses/by/4.0/ This content is distributed under the terms of the Creative Commons Attribution 4.0 International license.

### Combining partially functional canonical and noncanonical alleles leads to synthetic lethal phenotypes.

**FIG 8:**
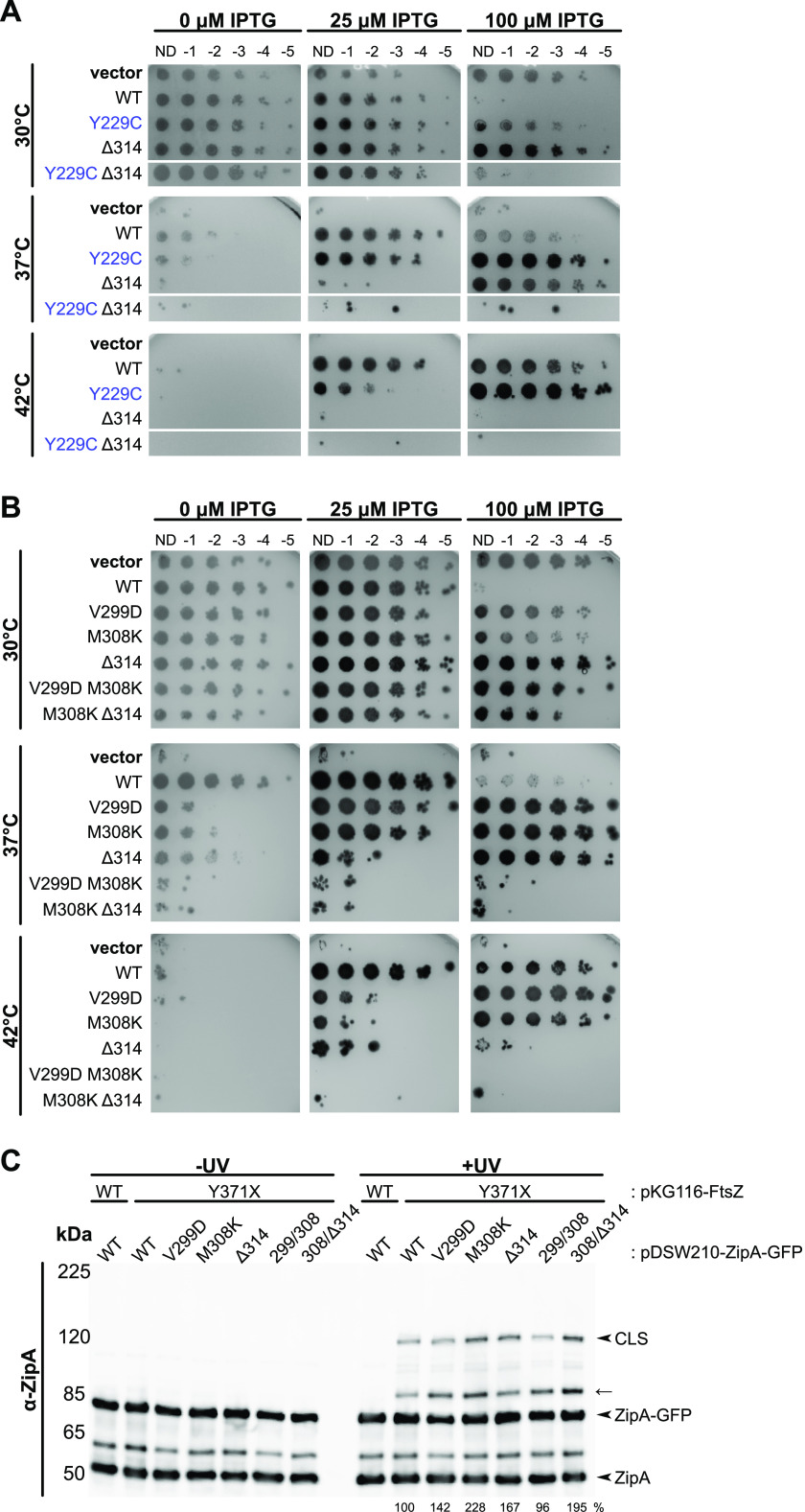
Combining single residue changes in noncanonical binding residues of ZipA decreases ZipA function. Cultures of strain WM5337 (*zipA1*) transformed with pDSW210-GFP (vector), pDSW210-ZipA-GFP, or derived plasmids carrying *zipA* mutations were serially diluted and spotted on plates containing 0, 25, or 100 μM IPTG and then grown at permissive (30°C) or restrictive (37°C and 42°C) temperatures. (A) ZipA-GFP carrying both canonical (Y229C) and noncanonical (ΔR314) residue changes failed to complement *zipA1* at restrictive temperatures. Panels shows composite images of two plates. (B) Combining mild noncanonical face mutations V299D and M308K together or with ΔR314 severely reduced ZipA-GFP function. (C) Bpa cross-linking in strain WM1074 carrying pUltra-pBpF, pKG116-FtsZ, and pDSW210-ZipA-GFP or derived constructs containing indicated mutations was assessed by Western blotting as described, except that cultures were induced with 25 μM IPTG. The FtsZ(Y371X) CTP maintained strong cross-linking with both single and double noncanonical site mutations. The arrow indicates probable CLS formed between FtsZ(Y371X) and native ZipA. Cross-linking efficiency relative to FtsZ(Y371X) is indicated below +UV lanes and was normalized by total protein levels and ZipA-GFP expression for each strain.

In addition to our screen for nontoxic ZipA mutants, we reasoned that one of the four residue changes comprising the thermosensitive *zipA1* allele, Y229C (10), might lead us to a useful loss-of-function mutant, as it is immediately adjacent to residues M226 and I228 that directly contact the FtsZ CTP in the cocrystal (Fig. S3) (16). We found that it conferred a mild defect, needing higher expression levels to complement the *zipA1* allele at 42°C than the WT protein (Fig. 8). Consistent with its ability to function in cell division at most temperatures, ZipA(Y229C)-GFP, like ZipA(V249E)-GFP, cross-linked robustly with FtsZ(Y371X) (Fig. 6). It could also cross-link to FtsZ well when combined with R314X but with reduced efficiency when combined with M226X (data not shown). In addition, ZipA(Y229C)-GFP localized to Z rings in *ΔzipA* cells (Fig. S2). From these data, we hypothesize that ZipA(Y229C) is partially defective due to perturbation of canonical binding.

10.1128/mbio.02529-21.3 FIG S3 E. coli ZipA amino acid sequence. Residues that form the *zipA1* mutant (blue), contact the FtsZ CTP in the crystal structure (L. Mosyak, Y. Zhang, E. Glasfeld, S. Haney, M. Stahl, J. Seehra, and W. S. Somers, EMBO J 19:3179−3191, 2000, https://doi.org/10.1093/emboj/19.13.3179) (green), or exhibit strong cross-linking with FtsZ (purple) are highlighted. Download FIG S3, PDF file, 0.1 MB. Copyright © 2021 Cameron et al. 2021 Cameron et al. https://creativecommons.org/licenses/by/4.0/ This content is distributed under the terms of the Creative Commons Attribution 4.0 International license.

Given the partially defective phenotypes of both, we asked whether combining the noncanonical mutant ΔR314 with the canonical mutant Y229C on the same molecule might result in a synthetic lethal phenotype if both types of FtsZ interactions were affected simultaneously. ZipA(Y229C)-GFP failed to complement the *zipA1* allele only at low induction levels (25 μM IPTG) at 42°C, and ZipA(ΔR314)-GFP was thermosensitive at all induction levels at 42°C and low induction levels at 37°C. In contrast, the double mutant failed to support growth of *zipA1* cells at any induction level at 42°C and 37°C (Fig. 8A) and even at 34°C (data not shown). This synthetic phenotype was probably not due to general instability of the protein, as the double mutant protein was toxic at the permissive temperature, comparable to WT ZipA (Fig. 8A). These results suggest that two separate binding activities of ZipA—canonical and noncanonical—contribute to ZipA function.

We next tested whether combining the ΔR314 mutant with the mild canonical mutant V249E would also result in a synthetic lethal phenotype. Indeed, compared to the single mutants, ZipA(V249E,ΔR314) was less effective at complementing *zipA1* under several conditions (Fig. S5), particularly at higher induction levels at 37°C. Again, combining canonical and noncanonical mutants resulted in a synthetic lethal phenotype, consistent with existence of two separate binding activities of ZipA.

10.1128/mbio.02529-21.5 FIG S5 Combining ZipA V249E and Δ314 mutations decreases *in vivo* function. Cultures of strain WM5337 (*zipA1*) transformed with pDSW210-GFP (vector), pDSW210-ZipA-GFP, or derived plasmids carrying *zipA* mutations were serially diluted, spotted on plates containing 0, 25, or 100 μM IPTG, and then grown at permissive (30°C) or restrictive (37°, 42°C) temperature. Cells expressing ZipA-GFP carrying the mild canonical face mutation V249E and the noncanonical ΔR314 mutation exhibited decreased viability at restrictive temperatures. Download FIG S5, PDF file, 0.1 MB. Copyright © 2021 Cameron et al. 2021 Cameron et al. https://creativecommons.org/licenses/by/4.0/ This content is distributed under the terms of the Creative Commons Attribution 4.0 International license.

### Double mutations in the noncanonical site confer severe defects.

Although we identified a single canonical site mutant, F269S, sufficient to completely disrupt ZipA function, no single noncanonical site mutant was equally defective. If noncanonical interactions occur over a larger region than canonical interactions, they may be less susceptible to complete disruption by single amino acid changes. Therefore, we tested whether combining multiple noncanonical mutant residues would have a greater impact on ZipA-GFP function. Indeed, when V299D and M308K or M308K and ΔR314 were combined, the resulting strains completely lost viability at all induction levels at 42°C and were nearly inviable at 37°C (Fig. 8B). The double mutant constructs were no longer able to complement the *zipA1* strain even at higher induction levels, unlike the individual single mutants. As with ZipA(F269S)-GFP, attempts to transduce Δ*zipA*::*kan* into a strain expressing ZipA(M308K,ΔR314)-GFP were unsuccessful, although this was possible with ZipA(V299D,M308K). Importantly, when FtsZ(Y371X) was used to measure canonical binding between ZipA and the FtsZ CTP, neither ZipA-GFP double mutant exhibited a decrease in CLS formation compared to ZipA-GFP (Fig. 8C). These data suggest that the double mutations within the noncanonical region weaken ZipA activity by interfering directly with noncanonical cross-linking to the FtsZ globular domain, and not through allosteric effects on canonical cross-linking to the FtsZ CTP.

### Alteration of a distal residue acts as an intragenic suppressor of the key canonical residue F269S.

**FIG 9:**
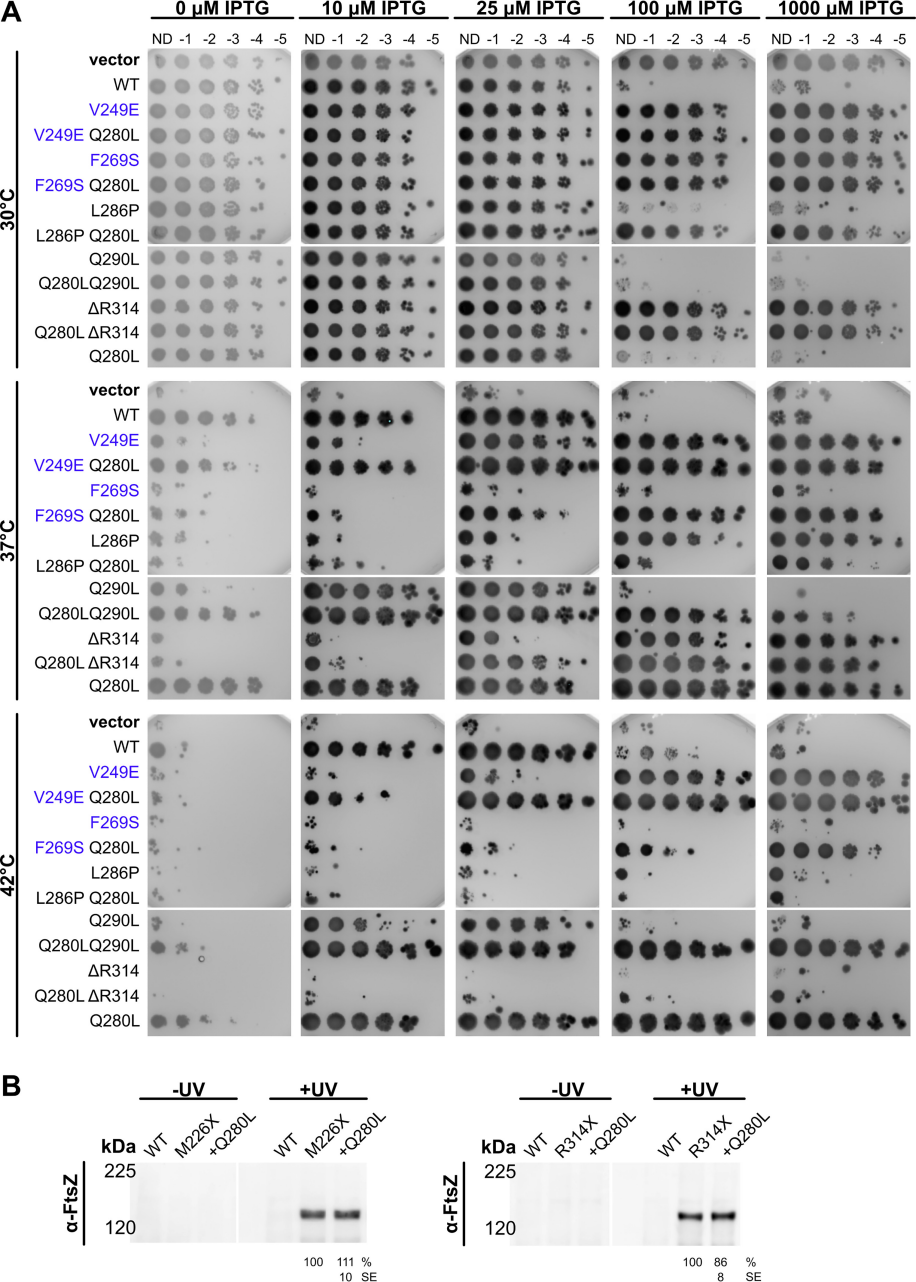
ZipA Q280L is an intragenic suppressor of FtsZ-binding mutants. (A) Cultures of strain WM5337 (*zipA1*) transformed with pDSW210-GFP (vector), pDSW210-ZipA-GFP, or derived plasmids carrying *zipA* mutations were serially diluted, spotted on plates containing 0, 10, 25, 100, or 1,000 μM IPTG, and then grown at permissive (30°C) or restrictive (37°, 42°C) temperatures. The Q280L mutation partially restored ZipA function in combination with the V249E, F269S, and Q290L mutations. Panels show composite images of two plates. (B) Cell lysates of strain WM1074 carrying pUltra-pBpF, pKG110-FtsZ, and pDSW210-ZipA-GFP or derived constructs containing indicated mutations were assessed by Western blotting in triplicate. The Q280L mutation did not significantly affect cross-linking at either the canonical (M226X) or noncanonical (R314X) sites. The mean and standard error (SE) of cross-linking efficiency relative to the control are indicated below the +UV lanes. Values were normalized by total protein levels and ZipA-GFP expression for each strain and then averaged across three replicates. Each panel is a composite image of a single representative blot (replicates not shown).

As part of our screen for mutants of ZipA that were less toxic than WT ZipA, we isolated Q280L, which is located near the noncanonical interface, 11 residues away from the key canonical residue F269. We noticed that in the absence of inducer, ZipA(Q280L) could complement the *zipA1* allele more efficiently (∼10-fold more viability) than WT ZipA (Fig. 9) and was also less toxic than WT ZipA under most conditions, suggesting that Q280L might be a hypermorphic allele and might compensate for other defects in ZipA interactions with FtsZ. To address this possibility, we combined Q280L with the severely defective canonical interaction allele F269S and tested its ability to complement *zipA1*.

Strikingly, ZipA(F269S,Q280L)-GFP showed full viability at 42°C at 1,000 μM IPTG and partial viability at 100 μM IPTG (Fig. 9A), in contrast to the complete lack of viability of ZipA(F269S)-GFP at either induction level. Similarly, whereas F269S allowed no viability at 37°C, the F269S Q280L double mutant was fully viable at or above 100 μM IPTG and partially viable even at 25 μM inducer (Fig. 9A). Unlike a strain expressing ZipA(F269S)-GFP, a strain expressing ZipA(F269S,Q280L)-GFP could be successfully transduced with Δ*zipA*::*kan*, and growth of the transductants was dependent on IPTG (Fig. S6), indicating that the Q280L allele was able to suppress the lethal phenotype of ZipA(F269S). Q280L also increased the viability of another mutant in the canonical site, ZipA(V249E). By itself, ZipA(V249E) was not viable with low (25 μM IPTG) induction at 37°C or 42°C. However, the ZipA(V249E,Q280L) double mutant became fully viable at this induction level and temperature, indicating that Q280L rescues both mild and severe deficiencies in ZipA’s canonical interactions with FtsZ. Notably, Q280L did not significantly rescue the noncanonical defects caused by L286P or ΔR314 (Fig. 9A), although it allowed Q290L to complement in a wider range of IPTG concentrations.

10.1128/mbio.02529-21.6 FIG S6 Q280L rescues viability of ZipA(F269S) in the absence of chromosomal *zipA*. WM5337 derivatives carrying either pWM6554 [ZipA(F269S)-GFP] or pWM6804 [ZipA(F269S,Q280L)-GFP] were transduced with *ΔzipA*::*aph* and selected on LB plates supplemented with kanamycin and IPTG. (A and B) Representative views of transduction plates. (C and D) Growth of *ΔzipA*::*aph* transductants carrying pWM6804 was dependent on IPTG. Download FIG S6, PDF file, 0.2 MB. Copyright © 2021 Cameron et al. 2021 Cameron et al. https://creativecommons.org/licenses/by/4.0/ This content is distributed under the terms of the Creative Commons Attribution 4.0 International license.

Finally, we asked whether Q280L caused differences in cross-linking with FtsZ. When combined with M226X or R314X, Q280L did not significantly affect canonical or noncanonical cross-linking (Fig. 9B), distinct from the greatly reduced cross-linking conferred by noncanonical changes Q290L or V299D. Q280L also did not enhance ZipA-FtsZ interactions in the yeast two-hybrid system (Fig. S4). These results suggest that Q280L’s ability to rescue canonical site mutants is due to factors other than enhancing interactions with FtsZ.

## DISCUSSION

**FIG 10:**
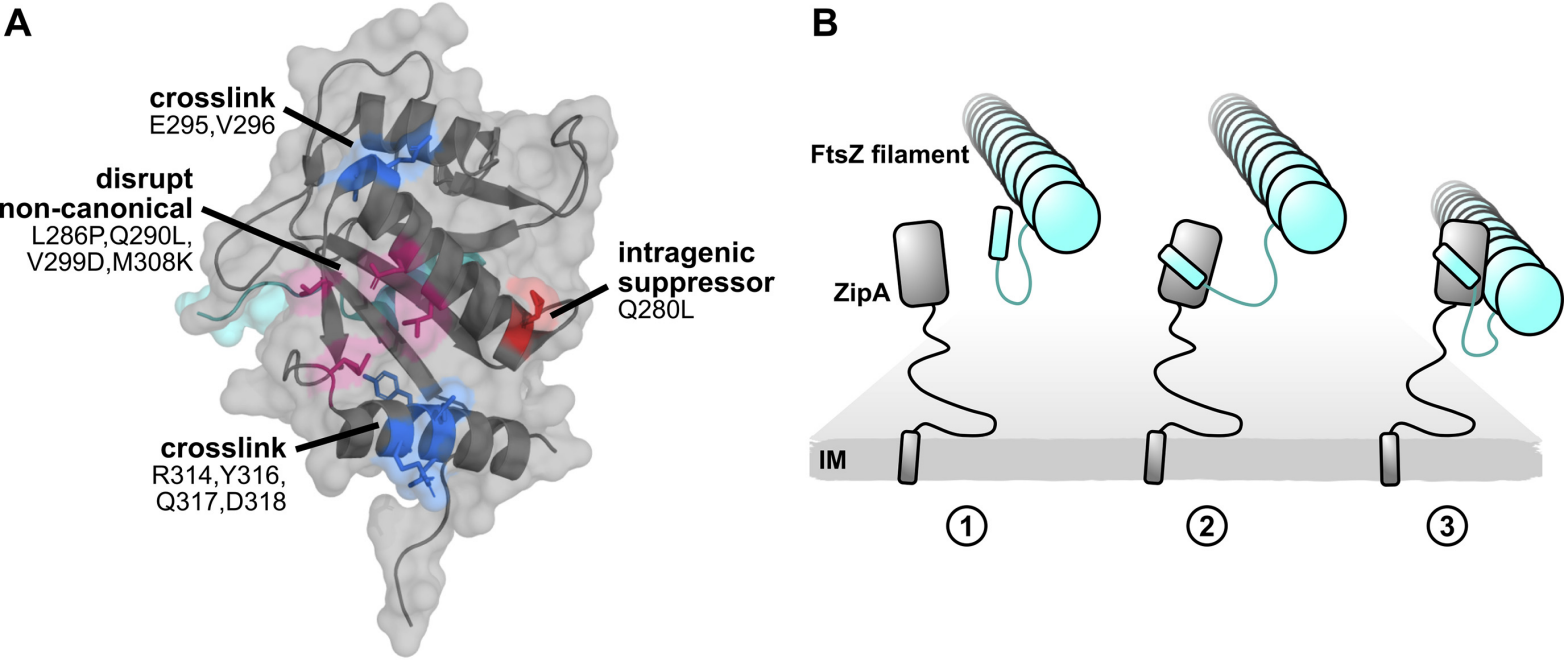
A model of ZipA-FtsZ binding and function. (A) ZipA crystal structure annotated with noncanonical cross-linking residues (blue), mutations that disrupt noncanonical cross-linking (magenta), and the intragenic suppressor Q280L (red). (B) A model illustrating how two-pronged binding might occur between ZipA and FtsZ. Here the FtsZ CTP first engages with the ZipA canonical binding site. This brings ZipA and FtsZ closer so that the ZipA noncanonical interface can fully engage with the FtsZ core domain. While only one ZipA was illustrated for simplicity, multiple proteins likely engage along the length of the FtsZ filament.

Here we have provided evidence that ZipA interacts with FtsZ through a previously unrecognized second binding site. Unlike the canonical FtsZ CTP-binding pocket in ZipA identified over 20 years ago (16), the noncanonical binding site in ZipA (Fig. 10A) interacts directly with the globular domain of FtsZ. As mutations introduced at either site on ZipA disrupt its function in cell division, both modes of binding are likely required for proper ZipA activity. However, it is not clear why ZipA would need to use multiple FtsZ-binding sites. One possible model is that ZipA initially contacts the FtsZ CTP, which in turn allows ZipA to bind to the FtsZ core subunit (Fig. 10B). This model would be consistent with the cryo-EM structure of full-length ZipA and FtsZ that places the globular domains of both proteins in close contact (20). Such a two-pronged binding model might also help explain why FtsA, which seemingly interacts only with the FtsZ CTP, has a lower binding affinity for FtsZ than ZipA.

Two FtsZ negative regulatory proteins, SlmA (23, 24) and MinC (25, 26), each also bind to both the FtsZ CTP and the FtsZ globular domain. In the model for MinC disruption of FtsZ rings, the interaction of the MinC C-terminal domain with the FtsZ CTP first brings the two proteins into close proximity, which facilitates binding between the N-terminal domain of MinC and the FtsZ globular domain that ultimately severs FtsZ polymers. Like MinC, SlmA binds strongly to the FtsZ CTP but also binds weakly to the FtsZ globular domain as suggested by structural studies (23) and specific mutations in the globular domain that confer resistance to SlmA toxicity (24). As a result, SlmA and MinC likely disrupt FtsZ polymerization through two independent binding events. Likewise, it is possible that ZipA engages FtsZ through two distinct binding events depending on its different activities at the Z ring. One event would be ZipA binding to the FtsZ CTP via canonical interactions while tethering treadmilling FtsZ polymers to the membrane. FtsA shares this activity, as it also binds to the FtsZ CTP. The second, more ZipA-specific activity would be its noncanonical binding to the FtsZ globular domain, which may promote a conformational change in FtsZ and/or interaction with FtsZ monomers. Unlike ZipA, FtsA is probably unable to provoke conformational changes in FtsZ through interaction with the FtsZ CTP alone. However, there is no established functional role for FtsZ monomer binding by ZipA, so it remains unclear whether disrupting this activity would lead to viability defects as observed with ZipA noncanonical mutants.

Alternatively, binding at both sites may be important for a different function of ZipA. The hypermorphic mutants FtsA* and FtsZ* that bypass the need for ZipA also promote FtsZ bundling (11, 27, 28). Consequently, the essential function of ZipA, so long as FtsA can serve as a membrane anchor for FtsZ, may be to stimulate FtsZ bundling by binding to FtsA (10) and breaking up FtsA minirings (11). If both FtsZ-binding sites are important for ZipA to facilitate FtsZ bundling, this might explain why most of the partially defective ZipA mutants described here still manage to localize to FtsZ rings. These mutants might disrupt FtsZ bundling through several mechanisms. As the FtsA interaction site on ZipA is unknown, this site may also be disrupted in the FtsZ-binding mutants. Alternatively, when bound to FtsZ at both sites, ZipA might be optimally oriented to disrupt FtsA minirings interacting with the FtsZ filament. Finally, although the available evidence argues against a direct FtsZ bundling role for ZipA (9), in such a model the two FtsZ-binding sites could presumably facilitate bundling by allowing ZipA to act as a bridge between multiple filaments. In this case, mutations disrupting either site could also prevent this function.

Although we have shown that there are two FtsZ-binding sites on ZipA, it is unclear to what degree these sites bind FtsZ independently. For example, loss-of-function mutations at either site in ZipA always resulted in some degree of disruption at the other site in cross-linking experiments using ZipA as the cross-linking donor. The interdependence between the two sites is also supported by genetic evidence, including decreased functionality after combining a weak canonical mutant with a weak noncanonical mutant, and the ability of Q280L near the ZipA noncanonical site to rescue several canonical site mutants. Future identification of residues in the FtsZ globular domain important for the noncanonical interaction as well as effects of ZipA mutations on FtsZ polymer dynamics and assembly state should greatly aid exploration of this question and those posed above.

## MATERIALS AND METHODS

### Strains, plasmids, and growth conditions.

All strains and plasmids used for this study are listed in Table S2 in the supplemental material. Bacterial cultures were grown with Lennox lysogeny broth (LB) or agar plates at 30°C unless otherwise indicated. Strains containing plasmids were supplemented as needed with ampicillin or carbenicillin (50 μg/ml), chloramphenicol (15 μg/ml), and spectinomycin (50 μg/ml). Optical density of liquid cultures was assessed at 600 nm (OD_600_).

10.1128/mbio.02529-21.9 TABLE S2 Strains and plasmids used in this study. Download Table S2, DOCX file, 0.03 MB. Copyright © 2021 Cameron et al. 2021 Cameron et al. https://creativecommons.org/licenses/by/4.0/ This content is distributed under the terms of the Creative Commons Attribution 4.0 International license.

### Construction of plasmids and strains.

To construct pUltra-pBpF, the *pbpf* gene was generated by PCR using primers CY179/CY180 and template pEvol-pbpf, and inserted into the pUltra vector using restriction enzyme NotI to provide pUltra-pBpF (29, 30). To generate pWM1350, the sequence for *zipA* corresponding to amino acids 189 to 328 was first amplified using primers 267/375, then digested with SacI and XbaI, and ligated into pCSK100 also cut with SacI and XbaI to create a ZipA(189-328)-GFP fusion. The resulting construct was digested with Ecl136II and SalI and then ligated with pLexA digested with SmaI and SalI, resulting in an in-frame LexA-ZipA-GFP fusion. All other plasmids were generated using site-directed mutagenesis (SDM) with the template and primers indicated in Table S2 and verified by DNA sequencing. Primer sequences are listed in Table S3. Complementation of *zipA* for most plasmid constructs was assessed by transducing WM5337 (*zipA1*) containing the plasmid of interest with P1 phage grown on WM1657 (*ΔzipA*::*aph*) and selecting for growth of kanamycin-resistant colonies in the presence of 25 μM IPTG. Complementation of ZipA(F269S,Q280L) was similarly tested; however, strain WM1074 containing the plasmid was transduced and plated with 500 μM IPTG instead. Δ*sulA*::*kan* derivatives of cross-linking strains were made by P1 transduction of the Δ*sulA*::*kan* Keio allele.

10.1128/mbio.02529-21.10 TABLE S3 Primers used in this study. Restriction sites or engineered mutations are capitalized. Download Table S3, DOCX file, 0.02 MB. Copyright © 2021 Cameron et al. 2021 Cameron et al. https://creativecommons.org/licenses/by/4.0/ This content is distributed under the terms of the Creative Commons Attribution 4.0 International license.

### Serial dilution plating assays.

Overnight cultures were diluted 1:200 in fresh medium and grown for 2 h to an OD_600_ of 0.2 to 0.4. Cultures were normalized by OD_600_ and then used to generate 10-fold serial dilutions. Dilutions were spotted onto LB agar plates containing the indicated concentrations of IPTG using a 48-pin cell replicator. Plates were incubated overnight at 30 to 42°C as indicated and then imaged for GFP fluorescence using a ChemiDoc MP imaging system (Bio-Rad) unless otherwise indicated.

### *In vivo* cross-linking.

Strain WM1074 was transformed with pUltra-pBpF and plasmids expressing FtsZ and ZipA constructs of interest. Overnight cultures were diluted 1:100 in 15 ml media and grown for 2.5 h. Unless noted otherwise, *p*-benzoyl-l-phenyl alanine (Bpa) and IPTG were added to a final concentration of 100 μM, and sodium salicylate was added to a concentration of 2 μM. Cultures were then grown in the dark for an additional 2.5 h. For each culture, the final OD_600_ was measured, and 10 ml was collected and pelleted at 4,000 × *g* for 10 min at 4°C. Pellets were resuspended in 2 ml of cold phosphate-buffered saline (PBS), then 1 ml was transferred to a 12-well microplate for UV exposure, and 1 ml was retained as the unexposed control.

A UVP 3UV lamp (Analytik Jena US) producing 1.57 mW/cm^2^ at 5 cm per manufacturer specifications was placed ∼2.5 cm over microplates to expose samples to 365-nm long-wave UV irradiation for 15 min at 4°C (estimated dose, ∼5.6 J/cm^2^). All samples were transferred to microtubes and pelleted at 16,000 × *g* for 2 min at room temperature (rt). Pellets were resuspended, normalized by OD_600_ in buffer containing 750 mM aminocaproic acid, 50 mM Bis-Tris, 2 mg/ml lysozyme, and cOmplete protease inhibitor (Roche), and then incubated on a rotator for 20 min at rt. Next sodium dodecyl sulfate (SDS) and *n*-dodecyl-beta-d-maltopyranoside (DDM) were added to a final concentration of 1%, and cells were incubated for an additional 20 min. Samples were subsequently subjected to three cycles of boiling for 5 min and freezing for 5 min and then centrifuged at 21,000 × *g* for 30 min at rt. Supernatant fractions were collected and mixed with 5× sample buffer (312 mM Tris [pH 6.8], 10% SDS, 50% glycerol, 25% beta-mercaptoethanol) to a final concentration of 2 × 10^6^ cells/μl (0.02 OD_600_/μl).

Although any SOS response in cells exposed to long-wave UV should be minimal (31), particularly with long-wavelength UV irradiation done at 4°C, we checked whether ZipA-FtsZ cross-linking or ZipA-GFP rings were affected by the cross-linking procedure in cells containing or lacking *sulA*, which is induced during SOS and causes Z-ring disassembly (32, 33). We found no significant effect of the presence of *sulA* on either canonical or noncanonical cross-linking, and ZipA-GFP rings were present in a similarly large proportion of *sulA*^+^ or Δ*sulA* cells after UV irradiation (see Fig. S7 in the supplemental material).

10.1128/mbio.02529-21.7 FIG S7 Exposure to 365-nm UV does not disrupt cell division. To test for possible UV-induced divisome disassembly mediated by SulA, *ΔsulA*::*kan* derivatives of cross-linking strains carrying pDSW210-ZipA-GFP with the R314X or M226X mutation were generated and assessed by Bpa cross-linking and microscopy. (A) Bpa-mediated cross-linking between ZipA-GFP and FtsZ was assessed in triplicate in strain WM1074 or strain WM1074 transduced with *ΔsulA*::*kan*, containing pUltra-pBpF, pKG110-FtsZ, and the indicated pDSW210-ZipA-GFP construct. Cell lysates were separated by 7.5% Tris-glycine SDS-PAGE, and cross-linking was compared between cells with or without *sulA*. The presence of *sulA* made no significant difference on Bpa cross-linking. The anti-FtsZ (α-FtsZ) blot shows a composite of two different exposures of the same blot. (B) ZipA-GFP localization was examined by fluorescence microscopy immediately following exposure to 365-nm UV for 15 minutes and compared to untreated controls. Images are representative fields of view. (C) Cells were randomly selected from differential interference contrast (DIC) images and scored for the presence or absence of a FtsZ ring in the corresponding fluorescence image. Neither UV treatment nor Δ*sulA*::*kan* resulted in a statistically significant difference in ZipA-GFP localization to mid-cell divisome rings. Download FIG S7, JPG file, 0.3 MB. Copyright © 2021 Cameron et al. 2021 Cameron et al. https://creativecommons.org/licenses/by/4.0/ This content is distributed under the terms of the Creative Commons Attribution 4.0 International license.

### Immunoblot analysis.

Samples were boiled for 10 min prior to separation by SDS-polyacrylamide gel electrophoresis (PAGE). Unless otherwise noted, 5 μl of each sample was separated using 5% Tris-glycine-asparagine-serine polyacrylamide gels (34) and Laemmli running buffer. Samples were transferred to nitrocellulose membranes using a Mini Trans-Blot apparatus (Bio-Rad) at 200 mA for 55 min in Towbin transfer buffer containing 0.1% SDS. Blots were stained by Ponceau staining to assess total protein levels, then blocked using 3% bovine serum albumin (BSA) in Tris-buffered saline with Tween 20 (TBST) and incubated with rabbit anti-FtsZ (1:3,000) or anti-ZipA (1:5,000) antibodies. After washes, blots were incubated with goat anti-rabbit antibody labeled with horseradish peroxidase (anti-rabbit-HRP) (1:10,000), developed with Pierce enhanced chemiluminescence (ECL) substrate and imaged using a ChemiDoc MP system.

### Yeast two-hybrid assays.

Untransformed Saccharomyces cerevisiae L40 was propagated in yeast extract-peptone-dextrose (YEPD) medium supplemented with 0.2% adenine and grown at 30°C. Yeast two-hybrid bait and prey plasmids were cotransformed into strain L40 using the lithium acetate/polyethylene glycol (PEG) protocol (35), and cotransformants were selected using SD -Trp/-Leu dropout medium (synthetic defined medium lacking tryptophan or leucine). Liquid β-galactosidase Miller assays were performed as described previously (35), except that colonies were taken directly from SD -Trp/-Leu plates after growth for 3 or 4 days and resuspended in media for use in the assay.

### Microscopy.

Overnight cultures were diluted 1:200 in liquid media containing 25 μM IPTG and grown for 1 h at 30°C at 250 rpm. Cultures were then split and continued at 30°C for 1 h or were shifted to a 42°C shaking water bath incubator for 1 h. Cells were mounted on agarose pads and imaged with an Olympus BX63 microscope with a Hamamatsu C11440 ORCA-Spark digital complementary metal oxide semiconductor (CMOS) camera using cellSens software (Olympus). Images were analyzed using Fiji/ImageJ.
