# Supplementary figures and images for: ZipA Uses a Two-Pronged FtsZ-Binding Mechanism Necessary for Cell Division
Source: mBio. 2021 Dec 14;12(6):e02529-21. doi: 10.1128/mbio.02529-21 (PMC8669495; doi:10.1128/mbio.02529-21)

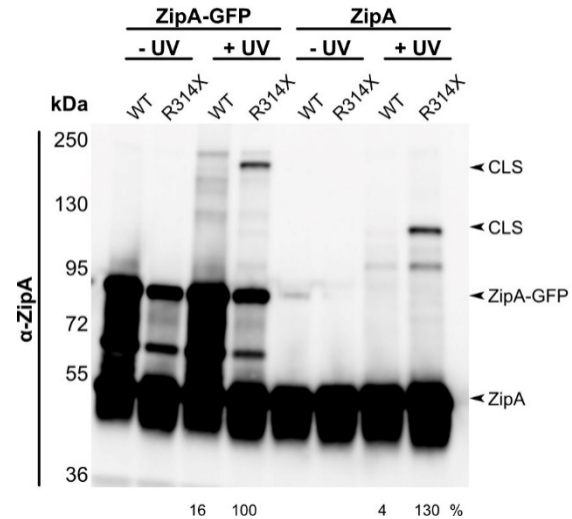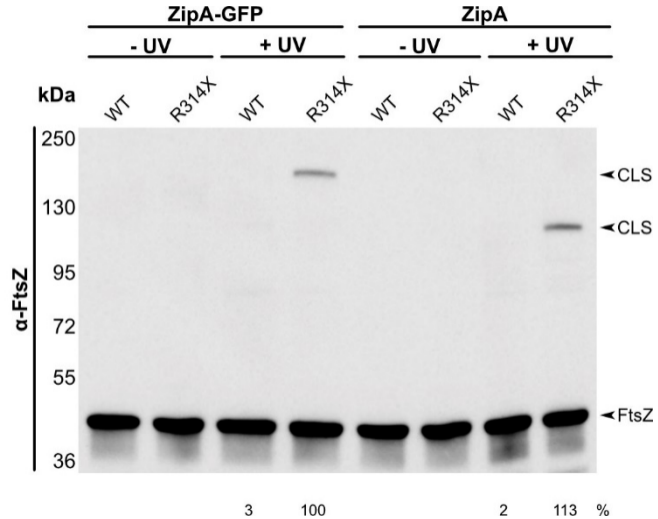

Supplement: FIG S1 [file mbio.02529-21-sf001.pdf]

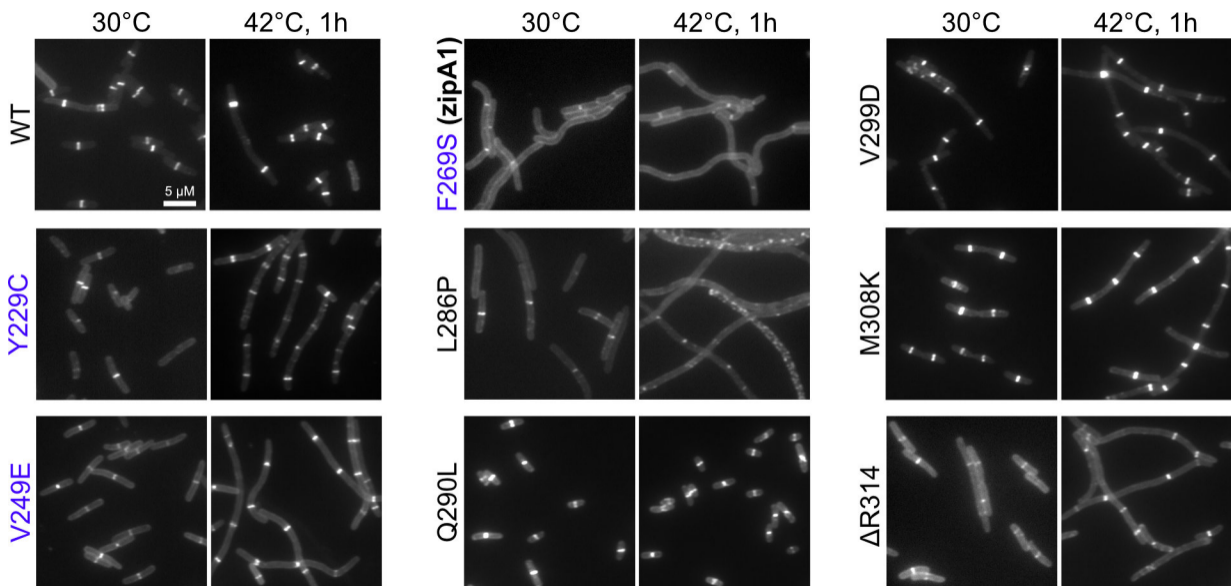

Supplement: FIG S2 [file mbio.02529-21-sf002.pdf]

Miller Units

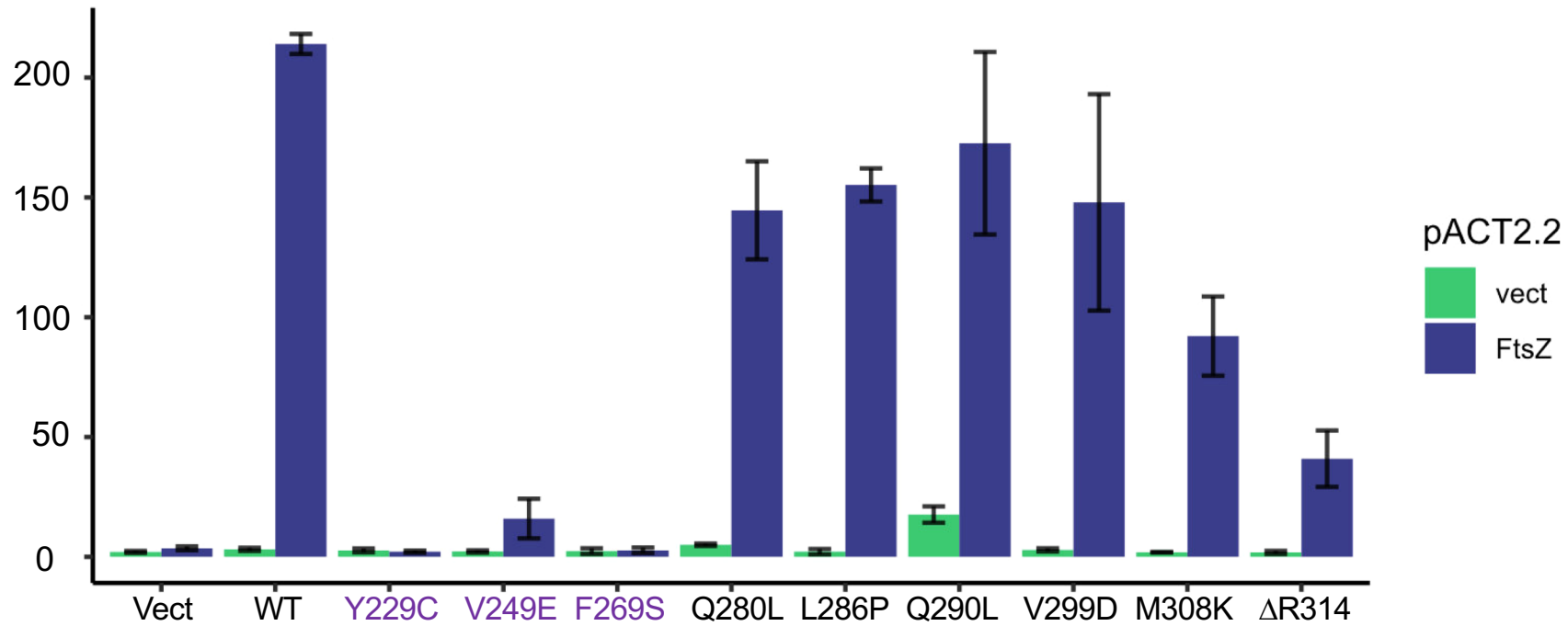

Supplement: FIG S4 [file mbio.02529-21-sf004.pdf]

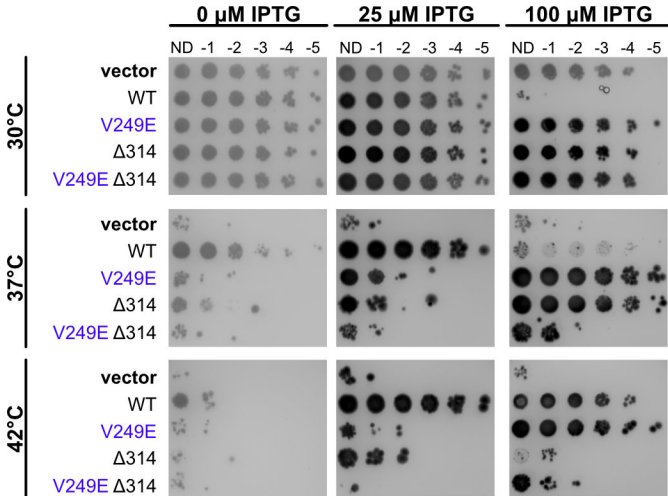

Supplement: FIG S5 [file mbio.02529-21-sf005.pdf]

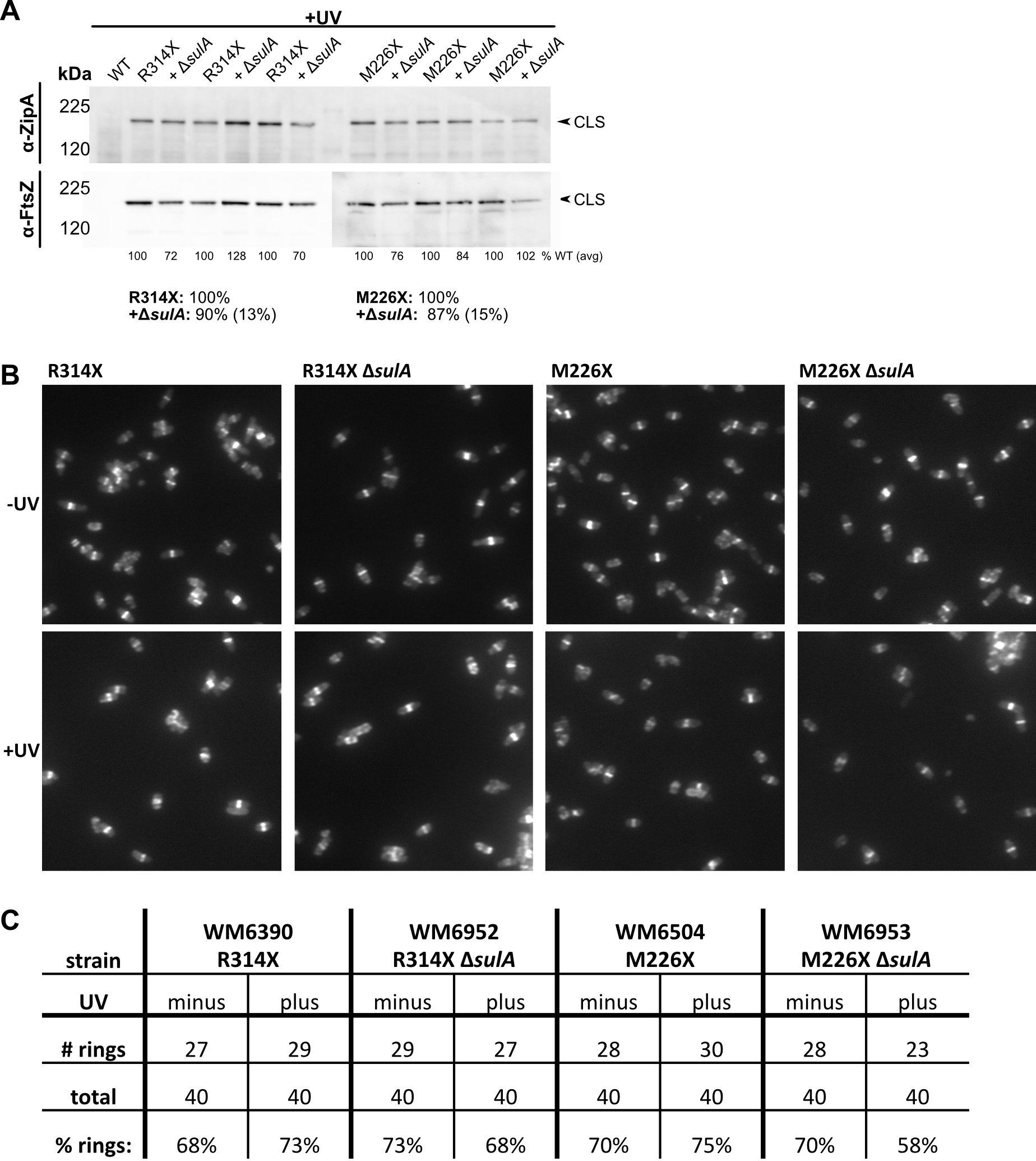

Supplement: FIG S7 [file mbio.02529-21-sf007.jpg]
