# Supplementary material for: ZipA Uses a Two-Pronged FtsZ-Binding Mechanism Necessary for Cell Division
Source: mBio. 2021 Dec 14;12(6):e02529-21. doi: 10.1128/mbio.02529-21 (PMC8669495; doi:10.1128/mbio.02529-21)
Supplement: TABLE S1 [file mbio.02529-21-st001.docx]

**Table S1.** ZipA mutations reconstructed for confirmatory analysis.

| **pDSW210-ZipA-GFP** | **interaction face** | **less toxic overexpression^a^** | **full *zipA1* complementation**^b^ |
| --- | --- | --- | --- |
| D225V | canonical proximal | N | Y |
| V249E | canonical | Y | N |
| D255V | canonical proximal | N | Y |
| F269S | canonical | Y | N |
| Q280L | non-canonical | Y | Y^c^ |
| F282S | non-canonical | N | Y |
| L286P | non-canonical | Y | N |
| Q290L | non-canonical | N | N |
| V299D | non-canonical | Y | N |
| D302A | canonical proximal | N | Y |
| M308K | non-canonical | Y | N |
| L313E | non-canonical | N | Y |

^a^compared to WT pDSW210-ZipA-GFP on serial dilution spot plates at 30°C with 100 μM IPTG

^b^at 42°C with 25 μM IPTG

^c^exceeded growth of WT pDSW210-ZipA-GFP by 1-2 fold with 0 μM IPTG
