# Supplementary material for: ZipA Uses a Two-Pronged FtsZ-Binding Mechanism Necessary for Cell Division
Source: mBio. 2021 Dec 14;12(6):e02529-21. doi: 10.1128/mbio.02529-21 (PMC8669495; doi:10.1128/mbio.02529-21)
Supplement: FIG S3 [file mbio.02529-21-sf003.pdf]

```
         10         20         30         40         50
MMQDLRLILI IVGAIAIIAL LVHGFWTSRK ERSSMFRDRP LKRMKSKRDD
         60         70         80         90        100
DSYDEDVEDD EGVGEVRVHR VNHAPANAQE HEAARPSPQH QYQPPYASAQ
        110        120        130        140        150
PRQPVQQPPE AQVPPQHAPH PAQPVQQPAY QPQPEQPLQQ PVSPQVAPAP
        160        170        180        190        200
QPVHSAPQPA QQAFQPAEPV AAPQPEPVAE PAPVMDKPKR KEAVIIMNVA
        210        220        230        240        250
AHHGSELNGE LLLNSIQQAG FIFGDMNIYH RHLSPDGSGP ALFSLANMVK
        260        270        280        290        300
PGTFDPEMKD FTTPGVTIFM QVPSYGDELQ NFKLMLQSAQ HIADEVGGVV
        310        320
LDDQRRMMTP QKLREYQDII REVKDANA
```

Highlighted are positions of M44V, L211T, Y229C, N281S (*zipA1* mutations). Note that Y229 is adjacent to a strong FtsZ contacting residue (I228)

Highlighted are residues in contact with FtsZ

Highlighted are residues that form strong crosslinking species containing FtsZ

TM is in orange
