# Supplementary material for: ZipA Uses a Two-Pronged FtsZ-Binding Mechanism Necessary for Cell Division
Source: mBio. 2021 Dec 14;12(6):e02529-21. doi: 10.1128/mbio.02529-21 (PMC8669495; doi:10.1128/mbio.02529-21)
Supplement: FIG S6 [file mbio.02529-21-sf006.pdf]

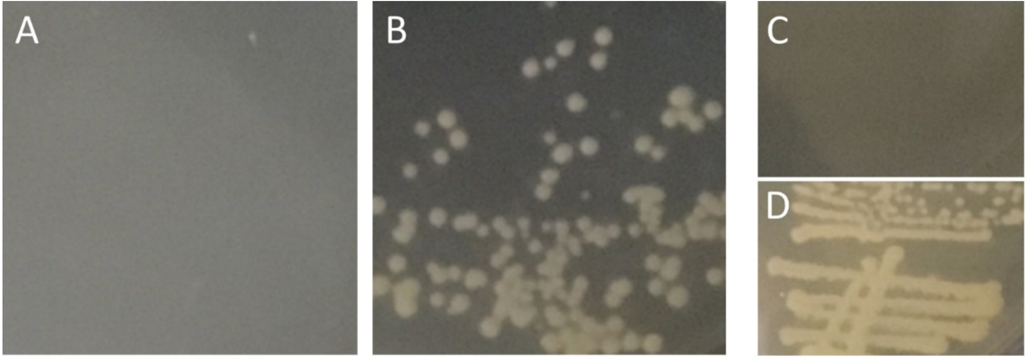

0 mM IPTG

0.5 mM IPTG

Δ*zipA*::*kan* transduced into pDSW210-*zipA(F269S)-GFP*

0.5 mM IPTG

Δ*zipA*::*kan* transduced into pDSW210-*zipA(F269S,Q280L)-GFP*

0.5 mM IPTG

Δ*zipA*::*kan* transduced into pDSW210-*zipA(F269S,Q280L)-GFP* *isolated transductant*
