# Supplementary material for: ZipA Uses a Two-Pronged FtsZ-Binding Mechanism Necessary for Cell Division
Source: mBio. 2021 Dec 14;12(6):e02529-21. doi: 10.1128/mbio.02529-21 (PMC8669495; doi:10.1128/mbio.02529-21)
Supplement: TABLE S2 [file mbio.02529-21-st002.docx]

**Table S2.** Strains and plasmids used in this study.

| **Strain** | **Description** | **Source*** |
| --- | --- | --- |
| JW0941 | Keio collection *ΔsulA::kan* | (1) |
| L40 | *S. cerevisiae* yeast two-hybrid *lexA lacZ* reporter strain | Lab collection |
| WM1074 | MG1655 *ΔlacU169* | Lab collection |
| WM1657 | *ftsA(R286W) zipA::aph (zipA::kan)* | (2) |
| WM5337 | MG1655 *zipA1 ΔnupC::tet* | (3) |
| WM6652 | MG1655 *zipA::aph pDSW210-ZipA-GFP* | this publication |
| WM6653 | MG1655 *zipA::aph pDSW210-ZipA(Y229C)-GFP* | this publication |
| WM6654 | MG1655 *zipA::aph pDSW210-ZipA(V249E)-GFP* | this publication |
| WM6655 | MG1655 *zipA::aph pDSW210-ZipA(L286P)-GFP* | this publication |
| WM6746 | MG1655 *zipA::aph pDSW210-ZipA(Q290L)-GFP* | this publication |
| WM6747 | MG1655 *zipA::aph pDSW210-ZipA(V299D)-GFP* | this publication |
| WM6748 | MG1655 *zipA::aph pDSW210-ZipA(M308K)-GFP* | this publication |
| WM6656 | MG1655 *zipA::aph pDSW210-ZipA(ΔR314)-GFP* | this publication |
| WM6861 | WM1074 *zipA::aph pDSW210-ZipA(F269S Q280L)-GFP* | this publication |
| WM6952 | WM1074 *∆sulA::kan* + pUltra-pBpF / pKG110-FtsZ / pDSW210-ZipA(R314X)-GFP | this publication |
| WM6953 | WM1074 *∆sulA::kan* + pUltra-pBpF / pKG110-FtsZ / pDSW210-ZipA(M226X)-GFP | this publication |
|  |  |  |
| **Plasmid** | **Description** | **Source** |
| pDSW210 | *colE1* plasmid with significantly weakened Ptrc promoter, amp^R^ | (4) |
| pKG110 | pACYC184 derivative containing the *nahG* promoter, cm^R^ | J.S. Parkinson |
| pKG116 | pKG110 derivative with stronger Shine-Dalgarno sequence, cm^R^ | J.S. Parkinson |
| pACT2.2 | yeast two-hybrid Gal4 activation fusion vector, amp^R^ Leu+ | S. Elledge, Baylor College of Medicine |
| pLexA | yeast two-hybrid LexA binding domain fusion vector, amp^R^ Trp+ | J. Jones, MD Anderson Cancer Center |
| pCSK100 | pBC (Stratagene) containing GFP M2 mutant cloned into BamHI site in the same frame as *lacZ* | (5) |
| pEvol-pBpF | pEvol containing tRNA synthetase/tRNA pair  for incorporation of p-benzoyl-L-phenylalanine | (6) |
| pUltra | CloDF13 (cdf) replicon compatible with p15a and colE1, spec^R^ | (7) |
| pUltra-pBpF | pUltra containing tRNA synthetase/tRNA pair  for incorporation of p-benzoyl-L-phenylalanine | this publication |
| pWM1209 | pACT2.2-FtsZ | (8) |
| pWM1350 | pLexA-ZipA-GFP | this publication |
| pWM2765 | pKG110-FtsZ (pDH156) | (9) |
| pWM2766 | pKG116-FtsZ (pDH161) | (9) |
| pWM5265 | pDSW210-ZipA-GFP | (3) |
| pWM5308 | pDSW210-ZipA(Y229C)-GFP | pWM5265 + SDM (DV1/2) |
| pWM6199 | pDSW210-ZipA(E295X)-GFP | pWM5265 + SDM (DV3/4) |
| pWM6200 | pDSW210-ZipA(V296X)-GFP | pWM5265 + SDM (DV5/6) |
| pWM6201 | pDSW210-ZipA(R314X)-GFP | pWM5265 + SDM (DV7/8 |
| pWM6203 | pDSW210-ZipA(Y316X)-GFP | pWM5265 + SDM (DV9/10) |
| pWM6204 | pDSW210-ZipA(Q317X)-GFP | pWM5265 + SDM (DV11/12) |
| pWM6205 | pDSW210-ZipA(D318X)-GFP | pWM5265 + SDM (DV13/14) |
| pWM6286 | pDSW210-ZipA(R314E)-GFP | pWM5265 + SDM (DV15/16) |
| pWM6287 | pDSW210-ZipA(R314W)-GFP | pWM5265 + SDM (DV17/18) |
| pWM6288 | pDSW210-ZipA(R314C)-GFP | pWM5265 + SDM (DV19/20) |
| pWM6289 | pDSW210-ZipA(R314P)-GFP | pWM5265 + SDM (DV21/22) |
| pWM6290 | pDSW210-ZipA(R314A)-GFP | pWM5265 + SDM (DV23/24) |
| pWM6291 | pDSW210-ZipA(ΔR314)-GFP | pWM5265 + SDM (DV25/26) |
| pWM6439 | pDSW210-ZipA(F269X)-GFP | pWM5265 + SDM (2282/3) |
| pWM6478 | pDSW210-ZipA | pWM5265 + SDM (2305/6) |
| pWM6481 | pDSW210-ZipA(R314X) | pWM6201 + SDM (2305/6) |
| pWM6495 | pDSW210-ZipA(M226X)-GFP | pWM5265 + SDM (2315/6) |
| pWM6496 | pDSW210-ZipA(I228X)-GFP | pWM5265 + SDM (2317/8) |
| pWM6497 | pDSW210-ZipA(M248X)-GFP | pWM5265 + SDM (2319/20) |
| pWM6498 | pDSW210-ZipA(K250X)-GFP | pWM5265 + SDM (2321/2) |
| pWM6499 | pDSW210-ZipA(Q271X)-GFP | pWM5265 + SDM (2323/4) |
| pWM6500 | pDSW210-ZipA(R305X)-GFP | pWM5265 + SDM (2325/6) |
| pWM6525 | pLexA-ZipA(Y229C)-GFP | pWM1350 + SDM (2350/1) |
| pWM6552 | pDSW210-ZipA(D225V)-GFP | pWM5265 + SDM (2369/70) |
| pWM6553 | pDSW210-ZipA(V249E)-GFP | pWM5265 + SDM (2371/2) |
| pWM6554 | pDSW210-ZipA(F269S)-GFP | pWM5265 + SDM (2373/4) |
| pWM6556 | pDSW210-ZipA(L313A)-GFP | pWM5265 + SDM (2377/8) |
| pWM6562 | pDSW210-ZipA(D302A)-GFP | pWM5265 + SDM (2375/6) |
| pWM6566 | pDSW210-ZipA(D255V)-GFP | pWM5265 + SDM (2379/80) |
| pWM6567 | pDSW210-ZipA(L286P)-GFP | pWM5265 + SDM (2381/2) |
| pWM6615 | pDSW210-ZipA(M226X V249E)-GFP | pWM6495 + SDM (2371/2) |
| pWM6616 | pDSW210-ZipA(R314X V249E)-GFP | pWM6201 + SDM (2371/2) |
| pWM6618 | pDSW210-ZipA(M226X F269S)-GFP | pWM6495 + SDM (2373/4) |
| pWM6619 | pDSW210-ZipA(R314X F269S)-GFP | pWM6201 + SDM (2373/4) |
| pWM6621 | pDSW210-ZipA(M226X L286P)-GFP | pWM6495 + SDM (2381/2) |
| pWM6622 | pDSW210-ZipA(R314X L286P)-GFP | pWM6201 + SDM (2381/2) |
| pWM6624 | pDSW210-ZipA(M226X ΔR314)-GFP | pWM6291 + SDM (2315/6) |
| pWM6626 | pDSW210-ZipA(Y229C ΔR314)-GFP | pWM6291 + SDM (2350/1) |
| pWM6660 | pKG116-FtsZ(Y371X) | pWM2766 + SDM (2406/2407) |
| pWM6684 | pLexA-ZipA(V249E)-GFP | pWM1350 + SDM (2371/2) |
| pWM6685 | pLexA-ZipA(L286P)-GFP | pWM1350 + SDM (2381/2) |
| pWM6686 | pLexA-ZipA(ΔR314)-GFP | pWM1350 + SDM (2422/3) |
| pWM6690 | pLexA-ZipA(F269S)-GFP | pWM1350 + SDM (2373/4) |
| pWM6726 | pDSW210-ZipA(F282S)-GFP | pWM5265 + SDM (2438/9) |
| pWM6727 | pDSW210-ZipA(Q290L)-GFP | pWM5265 + SDM (2446/7) |
| pWM6728 | pDSW210-ZipA(V299D)-GFP | pWM5265 + SDM (2442/3) |
| pWM6729 | pDSW210-ZipA(M308K)-GFP | pWM5265 + SDM (2444/5) |
| pWM6754 | pDSW210-ZipA(R314X Q280L)-GFP | pWM6201 + SDM (2436/7) |
| pWM6755 | pDSW210-ZipA(R314X Q290L)-GFP | pWM6201 + SDM (2446/7) |
| pWM6756 | pDSW210-ZipA(R314X V299D)-GFP | pWM6201 + SDM (2442/3) |
| pWM6757 | pDSW210-ZipA(R314X M308K)-GFP | pWM6201 + SDM (2444/5) |
| pWM6759 | pDSW210-ZipA(M226X Q280L)-GFP | pWM6495 + SDM (2436/7) |
| pWM6760 | pDSW210-ZipA(M226X Q290L)-GFP | pWM6495 + SDM (2446/7) |
| pWM6761 | pDSW210-ZipA(M226X V299D)-GFP | pWM6495 + SDM (2442/3) |
| pWM6762 | pDSW210-ZipA(M226X M308K)-GFP | pWM6495 + SDM (2444/5) |
| pWM6803 | pDSW210-ZipA(V249E Q280L)-GFP | pWM6772 + SDM (2371/2) |
| pWM6804 | pDSW210-ZipA(F269S Q280L)-GFP | pWM6772 + SDM (2373/4) |
| pWM6805 | pDSW210-ZipA(Q280L L286P)-GFP | pWM6772 + SDM (2381/2457) |
| pWM6806 | pDSW210-ZipA(Q280L Q290L)-GFP | pWM6772 + SDM (2446/7) |
| pWM6807 | pDSW210-ZipA(Q280L ΔR314)-GFP | pWM6772 + SDM (2422/3) |
| pWM6837 | pLexA-ZipA(Q280L)-GFP | pWM1350 + SDM (2436/7) |
| pWM6838 | pLexA-ZipA(Q290L)-GFP | pWM1350 + SDM (2446/7) |
| pWM6839 | pLexA-ZipA(V299D)-GFP | pWM1350 + SDM (2442/3) |
| pWM6840 | pLexA-ZipA(M308K)-GFP | pWM1350 + SDM (2444/5) |
| pWM6863 | pDSW210-ZipA(M308K ΔR314)-GFP | pWM6291 + SDM (2444/5) |
| pWM6864 | pDSW210-ZipA(V249E ΔR314)-GFP | pWM6553 + SDM (2422/3) |
| pWM6865 | pDSW210-ZipA(E295X ΔR314)-GFP | pWM6199 + SDM (2422/3) |
| pWM6868 | pDSW210-ZipA(V299D M308K)-GFP | pWM6729 + SDM (2442/3) |
| pWM6890 | pKG110-FtsZ(1-316) | pWM2765 + SDM (2475/6) |

*SDM = site-directed mutagenesis

**References**

1. Baba T, Ara T, Hasegawa M, Takai Y, Okumura Y, Baba M, Datsenko KA, Tomita M, Wanner BL, Mori H. 2006. Construction of Escherichia coli K-12 in-frame, single-gene knockout mutants: the Keio collection. Mol Syst Biol 2:2006.0008.

2. Geissler B, Elraheb D, Margolin W. 2003. A gain of function mutation in *ftsA* bypasses the requirement for the essential cell division gene *zipA* in *Escherichia coli*. Proc Natl Acad Sci USA 100:4197–4202.

3. Vega DE, Margolin W. 2018. Suppression of a thermosensitive zipA cell division mutant by altering amino acid metabolism. J Bacteriol 200:e00535-17.

4. Weiss DS, Chen JC, Ghigo JM, Boyd D, Beckwith J. 1999. Localization of FtsI (PBP3) to the septal ring requires its membrane anchor, the Z ring, FtsA, FtsQ, and FtsL. J Bacteriol 181:508–520.

5. Ma X, Ehrhardt DW, Margolin W. 1996. Colocalization of cell division proteins FtsZ and FtsA to cytoskeletal structures in living *Escherichia coli* cells by using green fluorescent protein. Proc Natl Acad Sci USA 93:12998–13003.

6. Young TS, Ahmad I, Yin JA, Schultz PG. 2010. An enhanced system for unnatural amino acid mutagenesis in *E. coli*. J Mol Biol 395:361–374.

7. Chatterjee A, Sun SB, Furman JL, Xiao H, Schultz PG. 2013. A versatile platform for single- and multiple-unnatural amino acid mutagenesis in *Escherichia coli*. Biochemistry 52:1828–1837.

8. Ma X, Margolin W. 1999. Genetic and functional analyses of the conserved C-terminal core domain of *Escherichia coli* FtsZ. J Bacteriol 181:7531–7544.

9. Haeusser DP, Rowlett VW, Margolin W. 2015. A mutation in *Escherichia coli ftsZ* bypasses the requirement for the essential division gene *zipA* and confers resistance to FtsZ assembly inhibitors by stabilizing protofilament bundling. Mol Microbiol 97:988–1005.
