# Supplementary material for: ZipA Uses a Two-Pronged FtsZ-Binding Mechanism Necessary for Cell Division
Source: mBio. 2021 Dec 14;12(6):e02529-21. doi: 10.1128/mbio.02529-21 (PMC8669495; doi:10.1128/mbio.02529-21)
Supplement: TABLE S3 [file mbio.02529-21-st003.docx]

**Table S3.** Primers used in this study. Restriction sites or engineered mutations are capitalized.

| **Primer** | **Description** | **Sequence** |
| --- | --- | --- |
| 267 | zipA XbaI rvs | caTCTAGAggcgttggcgtctttg |
| 375 | zipA SacI fwd | aaGAGCTCcaagcgcaaagaagcg |
| 2282 | zipA F269X fwd | gtcactatctAGatgcaggtacc |
| 2283 | zipA F269X rvs | acccggagtagtgaaatc |
| 2305 | pDSW210 3 fwd | taagcttggctgttttggc |
| 2306 | zipA 3 rvs | ggcgttggcgtctttgac |
| 2315 | zipA M226X fwd | ttttggcgatTAGaatatttaccatcgtc |
| 2316 | zipA M226X rvs | atgaagcccgcttgttga |
| 2317 | zipA I228X fwd | cgatatgaatTAGtaccatcgtcatcttagcc |
| 2318 | zipA I228X rvs | ccaaaaatgaagcccgct |
| 2319 | zipA M248X fwd | cctggcgaatTAGgtgaaaccggg |
| 2320 | zipA M248X rvs | ctgaataacgccgggccg |
| 2321 | zipA K250X fwd | gaatatggtgTAGccgggaacctttgatc |
| 2322 | zipA K250X rvs | gccaggctgaataacgcc |
| 2323 | zipA Q271X fwd | tatctttatgTAGgtaccgtcttac |
| 2324 | zipA Q271X rvs | gtgacacccggagtagtg |
| 2325 | zipA R305X fwd | tgacgatcagTAGcgtatgatgactccgcagaaattgc |
| 2326 | zipA R305X rvs | agcacgacaccgcccact |
| 2350 | zipA Y229C fwd | atgaatatttGccatcgtcatcttag |
| 2351 | zipA Y229C rvs | atcgccaaaaatgaagcc |
| 2369 | zipA D225V fwd | catttttggcGTTatgaatatttaccatc |
| 2370 | zipA D225V rvs | aagcccgcttgttgaatg |
| 2371 | zipA V249E fwd | ggcgaatatgGAGaaaccgggaac |
| 2372 | zipA V249E rvs | aggctgaataacgccggg |
| 2373 | zipA F269S fwd | tgtcactatcTCTatgcaggtac |
| 2374 | zipA F269S rvs | cccggagtagtgaaatcc |
| 2375 | zipA D302A fwd | cgtgcttgacGCTcagcgccgta |
| 2376 | zipA D302A rvs | acaccgcccacttcatcg |
| 2377 | zipA L313E fwd | tccgcagaaaGAGcgcgagtacc |
| 2378 | zipA L313E rvs | gtcatcatacggcgctga |
| 2379 | zipA D255V fwd | gggaacctttGTTcctgaaatgaag |
| 2380 | zipA D255V rvs | ggtttcaccatattcgcc |
| 2381 | zipA L286P fwd | caagctgatgCCGcaatctgcgc |
| 2381 | zipA L286P fwd | caagctgatgCCGcaatctgcgc |
| 2382 | zipA L286P rvs | aagttctgcagctcgtcac |
| 2382 | zipA L286P rvs | aagttctgcagctcgtcac |
| 2406 | ftsZ Y371X fwd | agagccggatTAGctggatatccc |
| 2407 | ftsZ Y371X rvs | ttcgcagtttgcggcgca |
| 2422 | zipA Δ314 fwd | gagtaccaggacatcatc |
| 2423 | zipA Δ314 rvs | caatttctgcggagtcatc |
| 2436 | zipA Q280L fwd | tgacgagctgCTTaacttcaagctg |
| 2437 | zipA Q280L rvs | ccgtaagacggtacctgc |
| 2438 | zipA F282S fwd | gctgcagaacTCGaagctgatgc |
| 2439 | zipA F282S rvs | tcgtcaccgtaagacggt |
| 2442 | zipA V299D fwd | agtgggcggtGATgtgcttgacg |
| 2443 | zipA V299D rvs | tcatcggcaatatgctgc |
| 2444 | zipA M308K fwd | gcgccgtatgAAAactccgcaga |
| 2445 | zipA M308K rvs | tgatcgtcaagcacgacac |
| 2446 | zipA Q290L fwd | caatctgcgcTgcatattgcc |
| 2447 | zipA Q290L rvs | cagcatcagcttgaagttc |
| 2457 | zipA L286P+Q280L rvs | aagttAAGcagctcgtcac |
| 2475 | ftsZ glob fwd | taactcgaggatccgcgg |
| 2476 | ftsZ glob rvs | catgccgatacctgtcga |
| CY179 | Pbpf-insert-fwd | aatttcacaaaggaggtgcggccgcatggacgaatttgaaatgataaagagaaacacatct |
| CY180 | Pbpf-insert-rvs | ggagaccgtttaaacgcggccgcttataatctctttctaattggctctaaaatctttataagttcttcag |
| DV1 | ZipA Y229C rvs | ctaagatgacgatgGCAaatattcatatcgccaaaaatgaagccc |
| DV2 | ZipA Y229C fwd | gggcttcatttttggcgatatgaatattTGCcatcgtcatcttag |
| DV3 | ZipA E295X rvs | gacaccgcccacCTAatcggcaatatgctgcgc |
| DV4 | ZipA E295X fwd | gcgcagcatattgccgatTAGgtgggcggtgtc |
| DV5 | ZipA V296X rvs | cgacaccgccCTAttcatcggcaatatgctgcgc |
| DV6 | ZipA V296X fwd | gcgcagcatattgccgatgaaTAGggcggtgtcg |
| DV7 | ZipA R314X rvs | cggatgatgtcctggtactcCTAcaatttctgcggagtcatca |
| DV8 | ZipA R314X fwd | tgatgactccgcagaaattgTAGgagtaccaggacatcatccg |
| DV9 | ZipA Y316X rvs | gatgatgtcctgCTActcgcgcaatttctgcgg |
| DV10 | ZipA Y316X fwd | ccgcagaaattgcgcgagTAGcaggacatcatc |
| DV11 | ZipA Q317X rvs | tcgcggatgatgtcCTAgtactcgcgcaatttc |
| DV12 | ZipA Q317X fwd | gaaattgcgcgagtacTAGgacatcatccgcga |
| DV13 | ZipA D318X rvs | ctttgacttcgcggatgatCTActggtactcgcgcaatttc |
| DV14 | ZipA D318X fwd | gaaattgcgcgagtaccagTAGatcatccgcgaagtcaaag |
| DV15 | ZipA R314E rvs | ggatgatgtcctggtactcCTCcaatttctgcggagtcatc |
| DV16 | ZipA R314E fwd | gatgactccgcagaaattgGAGgagtaccaggacatcatcc |
| DV17 | ZipA R314W rvs | gatgatgtcctggtactcCCAcaatttctgcggagtcat |
| DV18 | ZipA R314W fwd | atgactccgcagaaattgTGGgagtaccaggacatcatc |
| DV19 | ZipA R314C rvs | tgtcctggtactcGCAcaatttctgcggagtc |
| DV20 | ZipA R314C fwd | gactccgcagaaattgTGCgagtaccaggaca |
| DV21 | ZipA R314P rvs | atgtcctggtactcGGGcaatttctgcggag |
| DV22 | ZipA R314P fwd | ctccgcagaaattgCCCgagtaccaggacat |
| DV23 | ZipA R314A rvs | gatgtcctggtactcGGCcaatttctgcggagtca |
| DV24 | ZipA R314A fwd | tgactccgcagaaattgGCCgagtaccaggacatc |
| DV25 | ZipA ΔR314 rvs | atgatgtcctggtactccaatttctgcggagtca |
| DV26 | ZipA ΔR314 fwd | tgactccgcagaaattggagtaccaggacatcat |
